# Collaborative development of predictive toxicology applications

**DOI:** 10.1186/1758-2946-2-7

**Published:** 2010-08-31

**Authors:** Barry Hardy, Nicki Douglas, Christoph Helma, Micha Rautenberg, Nina Jeliazkova, Vedrin Jeliazkov, Ivelina Nikolova, Romualdo Benigni, Olga Tcheremenskaia, Stefan Kramer, Tobias Girschick, Fabian Buchwald, Joerg Wicker, Andreas Karwath, Martin Gütlein, Andreas Maunz, Haralambos Sarimveis, Georgia Melagraki, Antreas Afantitis, Pantelis Sopasakis, David Gallagher, Vladimir Poroikov, Dmitry Filimonov, Alexey Zakharov, Alexey Lagunin, Tatyana Gloriozova, Sergey Novikov, Natalia Skvortsova, Dmitry Druzhilovsky, Sunil Chawla, Indira Ghosh, Surajit Ray, Hitesh Patel, Sylvia Escher

**Affiliations:** 1 Douglas Connect, Baermeggenweg 14, 4314 Zeiningen, Switzerland; 2 In silico Toxicology, Altkircher Str. 4 CH-4052 Basel, Switzerland; 3 Ideaconsult Ltd, A. Kanchev 4, Sofia 1000, Bulgaria; 4 Istituto Superiore di Sanità, Environment and Health Department, Istituto Superiore di Sanita', Viale Regina Elena 299, Rome 00161, Italy; 5 Technical University of Munich, Technische Universität München, Arcisstr. 21, 80333 München, Germany; 6 Albert-Ludwigs University Freiburg, 79110 Freiburg i.Br., Germany; 7 National Technical University of Athens, School of Chemical Engineering, Heroon Polytechneiou 9, 15780, Zographou, Athens, Greece; 8 David Gallagher, Congresbury, Somerset, UK; 9 Institute of Biomedical Chemistry of Russian Academy of Sciences, 119121 Moscow, Russia; 10 Seascape Learning, 271 Double Story, New Rajinder Ngr., New Delhi 110060, India; 11 Jawaharlal Nehru University, New Mehrauli Road, New Delhi 110067, India; 12 Fraunhofer Institute for Toxicology & Experimental Medicine, Nikolai-Fuchs-Str. 1, 30625 Hannover, Germany

## Abstract

OpenTox provides an interoperable, standards-based Framework for the support of predictive toxicology data management, algorithms, modelling, validation and reporting. It is relevant to satisfying the chemical safety assessment requirements of the REACH legislation as it supports access to experimental data, (Quantitative) Structure-Activity Relationship models, and toxicological information through an integrating platform that adheres to regulatory requirements and OECD validation principles. Initial research defined the essential components of the Framework including the approach to data access, schema and management, use of controlled vocabularies and ontologies, architecture, web service and communications protocols, and selection and integration of algorithms for predictive modelling. OpenTox provides end-user oriented tools to non-computational specialists, risk assessors, and toxicological experts in addition to Application Programming Interfaces (APIs) for developers of new applications. OpenTox actively supports public standards for data representation, interfaces, vocabularies and ontologies, Open Source approaches to core platform components, and community-based collaboration approaches, so as to progress system interoperability goals.

The OpenTox Framework includes APIs and services for compounds, datasets, features, algorithms, models, ontologies, tasks, validation, and reporting which may be combined into multiple applications satisfying a variety of different user needs. OpenTox applications are based on a set of distributed, interoperable OpenTox API-compliant REST web services. The OpenTox approach to ontology allows for efficient mapping of complementary data coming from different datasets into a unifying structure having a shared terminology and representation.

Two initial OpenTox applications are presented as an illustration of the potential impact of OpenTox for high-quality and consistent structure-activity relationship modelling of REACH-relevant endpoints: ToxPredict which predicts and reports on toxicities for endpoints for an input chemical structure, and ToxCreate which builds and validates a predictive toxicity model based on an input toxicology dataset. Because of the extensible nature of the standardised Framework design, barriers of interoperability between applications and content are removed, as the user may combine data, models and validation from multiple sources in a dependable and time-effective way.

## 1. Background

### 1.1 Introduction

In a study by the European Chemical Bureau (ECB), it was estimated that the new EU chemical legislation REACH would require 3.9 million additional test animals, if no alternative methods were accepted [1]. The same study showed that it was possible to reduce the number of test animals significantly by utilizing existing experimental data in conjunction with (Quantitative) Structure Activity Relationship ((Q)SAR) models. Chronic and reproductive toxicity, *in vivo *mutagenicity and carcinogenicity are the endpoints that will require the largest number of test animals within REACH, because no alternative *in vitro *assays are available yet.

Recent developments allow a more accurate prediction of complex toxicological endpoints than a few years ago. This progress has been supported by (i) the development of improved (Q)SAR algorithms, (ii) the availability of larger and better curated public databases, (iii) progress in computational chemistry and biology, and (iv) the development of an array of *in vitro *assays probing targets, pathways and endpoints.

The routine application of these new generation models is however still rare, because

• Toxicity data has been collected in a variety of different databases;

• These databases use different formats, that are frequently not generally compatible with *in silico *programs;

• Many toxicity databases lack important information for modelling (e.g. curated chemical structures; inability to select and combine data from multiple sources);

• It is hard to integrate confidential in-house data with public data for model building and validation;

• Models have been published in a variety of different formats (ranging from simple regression based equations to full-fledged computer applications);

• There is no straightforward integration of predictions from various applications;

• There is no commonly accepted framework for the validation of *in silico *predictions and many *in silico *tools provide limited support for reliable validation procedures;

• The application, interpretation, and development of (Q)SAR models is still difficult for most toxicological experts. It requires a considerable amount of statistical, cheminformatics and computer science expertise and the procedures are labour-intensive and prone to human errors.

The EC-funded FP7 project "OpenTox" [2] aims to address these issues. The overall objective of OpenTox is to develop a framework that provides a unified access to *in vitro *and *in vivo *toxicity data, *in silico *models, procedures supporting validation and additional information that helps with the interpretation of predictions. OpenTox is accessible at three levels:

• A simplified user interface for toxicological experts that provides unified access to predictions, toxicological data, models and supporting information;

• A modelling expert interface for the streamlined development and validation of new models;

• Public OpenTox Application Programming Interfaces (APIs) for the development, integration and validation of new algorithms and models.

The core components of the OpenTox Framework are being developed or integrated with an open source licensing approach to optimize the dissemination and impact of the platform, to allow the inspection and review of algorithms, and to be open to potential contributions of value from the scientific community.

### 1.2 OpenTox Objectives

The overall long-term goal of OpenTox is the development of an interoperable, extensible predictive toxicology framework containing a collection of state-of-the art (Q)SAR, cheminformatics, bioinformatics, statistical and data mining tools, computational chemistry and biology algorithms and models, integratable *in vitro *and *in vivo *data resources, ontologies and user-friendly Graphical User Interfaces (GUIs). OpenTox supports toxicological experts without specialist *in silico *expertise as well as model and algorithm developers. It moves beyond existing attempts to create individual research resources and tools, by providing a flexible and extensible framework that integrates existing solutions and new developments.

### 1.3 OpenTox Design Principles

The design principles of interoperability, flexibility, transparency and extensibility are key ingredients of the OpenTox Framework design, which additionally guide its architecture and implementation.

#### 1.3.1 Interoperability

Interoperability with respect to the OpenTox Framework refers to the principle that different OpenTox components or services may correctly exchange information with each other and subsequently make use of that information. Both syntactic interoperability for correct data exchange and semantic interoperability supporting the accurate communication of meaning and interpretation of data are supported principles for OpenTox resources. The principles are reflected design-wise in the use of open, standardised interfaces and ontologies. The principles are relevant in application development and deployment when a combination of distributed multiple services can provide value to a user in completing a use case satisfactorily.

#### 1.3.2 Flexibility

As a significant variety of user scenarios, requirements and use cases in predictive toxicology exist, flexibility is a key principle incorporated into OpenTox. Through the use of a component-based approach and the incorporation of the interoperability principles, many different and customised applications can be assembled that are based on the underlying platform.

#### 1.3.3 Transparency

To achieve the scientific objective of knowledge-based enquiry based on principles of reasoning, reproducibility, and reliability, OpenTox supports the principle of transparency in its design. Computational models should be available for scrutiny by other scientists in as complete a manner and detail as possible. Evaluators and regulators should be able to both understand the details and accurately reproduce the results of predictive toxicity models, and be able to reliably form judgements on their validity as evidence. The principle also supports achievement of the OECD validation principles such as an unambiguous algorithm and a mechanistic interpretation, if possible. Use of Open Source, Open Interfaces and Standards within OpenTox support implementation of the transparency principle applied to *in silico*-based predictive toxicology applications and their reported results.

#### 1.3.4 Extensibility

The field of predictive toxicology is rapidly developing and broadening in many areas including the use of biomarkers, systems biology, epigenetics, toxicokinetics, *in vitro *assays, stem cell technology, and computational chemistry and biology. Hence, OpenTox needs to be extensible to a broad range of future predictive toxicology applications. In such applications, contributing and diverse experimental data and models need to be combined as evidence supporting integrated testing, safety and risk assessment and regulatory reporting as stipulated under REACH. In the initial design of the OpenTox Framework we have first attempted to create a general solution for (Q)SAR model development and application. We also will address and strengthen its extensibility in subsequent extensions of the OpenTox APIs, and guided by suitable use cases, to additional areas of scientific enquiry in the predictive toxicology field as part of its evolutionary development.

### 1.4 Toxicity Data

Toxicity data has been traditionally dispersed over a variety of databases where only a small fraction was immediately suitable for *in silico *modelling and structure-based searches because they contained chemical structures and defined toxicological endpoints. Recent efforts (e.g. from Istituto Superiore di Sanità (ISS), Fraunhofer Institute for Toxicology & Experimental Medicine (FhG ITEM), US Environmental Protection Agency (US EPA), US Food & Drug Administration (US FDA)) have improved the situation, because they provide curated data that has been compiled from various sources (public testing programs, general literature, non-confidential in-house data). Public repositories of bioassay data like PubChem [3] provide additional information that can be used for toxicological risk assessment.

The aggregation of data from different sources is however still far from trivial and poses some interesting toxicological, computer science, technological and legal questions, e.g.:

• Reliable identification of database entries that point to identical primary experiments;

• Reliable mapping from various non-unique chemical identifiers (e.g. names, CAS numbers) to chemical structures;

• Development of ontologies that describe the relationships between the various toxicological effects and mechanisms and related chemical and biological entities;

• Utilization of high content and high throughput screening data for toxicity predictions;

• Integration of databases with different access policies (and legal status);

• Structure anonymisation to share toxicity data from sensitive in-house datasets (if possible [4]);

• Systematic data quality assessment.

As the size of toxicity databases prohibits a manual inspection of all data, it is necessary to apply advanced data- and text-mining techniques to solve most of these tasks automatically and to identify instances that need human inspection.

Some of the data integration issues have already been addressed by other computational toxicology and chemistry initiatives e.g. ECB QSAR Model Reporting Format [5], DSSTox [6], ToxML [7], CDK [8], InChI [9]. However although these approaches solve some technical aspects of data integration, none of them provides an architecture for the seamless merging and use of toxicity data from various sources. An OpenTox goal is to provide unified access to existing tools for data integration, develop new tools for this purpose, provide sound validation techniques and aid driving efforts to develop standards in this area.

### 1.5 Ontologies

The definition of ontology and controlled vocabulary in OpenTox is required so as to standardize and organize high-level concepts, chemical information and toxicological data. Distributed OpenTox services exchanging communications need to have unambiguous interpretations of the meaning of any terminology and data that they exchange between each other.

Prioritisation of OpenTox toxicological endpoints focuses on those endpoints recognized internationally as critical for the testing of chemicals. Primary sources of information include the OECD guidelines for testing of chemicals [10,11] and the toxicological endpoints relevant to the assessment of chemicals in the EU [12].

*A further more detailed definition of Ontology in this context is provided in *Additional File 1.

### 1.6 Approach to Predictive Toxicology (Q)SARs

Initial OpenTox work has focused on creating a Framework for the support of (Q)SAR-based data driven approaches.

#### 1.6.1 Toxicity (Q)SARs

Because of its relevance for the reduction of animal testing, we are focusing initially on the reproductive toxicity, chronic toxicity, mutagenicity and carcinogenicity endpoints. The OpenTox Framework however works independently of the underlying data, which makes it useful also for any other toxicology-relevant endpoints.

The main problem for toxicological modellers is that they have to deal with endpoints with very complex and frequently unknown biological mechanisms and with datasets with very diverse structures. This currently prohibits in many cases a systems biology approach as well as the application of simple regression-based techniques. For this reason advanced data mining and cheminformatics techniques are gaining increasing acceptance within the toxicological community. Modern techniques like lazar [13], fminer [14] and iSAR [15] allow the automated determination of relevant chemical descriptors and the generation of prediction models that are understandable and interpretable by non-computer scientists.

Many (Q)SAR models for the prediction of mutagenic and carcinogenic properties have been developed in recent years. The prediction of bacterial mutagenicity is relatively successful (typical accuracies 80%), but the success with carcinogenicity predictions has been much more limited and very few models are available for *in vivo *mutagenicity. With recent developments like lazar, it is however possible to predict rodent carcinogenicity with accuracies similar to bacterial mutagenicity and to achieve a reliable estimation of prediction confidences. It is likely that further improvements can be obtained with better algorithms for chemical and biological feature generation, feature selection and model generation, and the novel combination of existing techniques.

#### 1.6.2 Aggregation of Predictions from various Models

It is known from machine learning, that the aggregation of different prediction models leads to increased accuracies [16]. The aggregation of predictions from different *in silico *programs is however still a cumbersome task that requires a lot of human intervention and *ad hoc *solutions. A new plugin-architecture is therefore needed that allows an easy integration of models and programs from different origins, independently of their programming language and legal status. Similar plugin facilities are needed for algorithms that perform a dedicated task during model generation (e.g. feature generation, feature selection, classification, regression). With such a modularized approach it will be easier to experiment with new algorithms and new combinations of algorithms and to compare the results with benchmarked methods.

#### 1.6.3 Validation of Models

An objective validation framework is crucial for the acceptance and the development of *in silico *models. The risk assessor needs reliable validation results to assess the quality of predictions; model developers need this information to (i) avoid the overfitting of models, (ii) to compare new models with benchmarked techniques and (iii) to get ideas for the improvement of algorithms (e.g. from the inspection of misclassified instances). Validation results can also be useful for data providers as misclassifications point frequently to flawed database entries. OpenTox is actively supporting the OECD Principles for (Q)SAR Validation so as to provide easy-to-use validation tools for algorithm and model developers.

Care must be taken, that no information from test sets leaks into the training set, either performing certain steps (frequently supervised feature generation or selection) for the complete dataset or by "optimizing" parameters until the resulting model fits a particular test set by chance. For this reason OpenTox provides standardized validation routines within the framework that can be applied to all prediction algorithms that are plugged into the system. These kinds of techniques are standard in the field of machine learning and data-mining, but are however not yet consistently employed within the field of (Q)SAR modelling.

#### 1.6.4 Determination of Applicability Domains

For practical purposes it is important to know the proportion of compounds that fall within the Applicability Domain (AD) of a certain model. For this purpose OpenTox will provide automated facilities to identify the proportion of reliable predictions for the "chemical universe" e.g. structures of the database [17], particular subsets (e.g. certain classes of pharmaceuticals, food additives, REACH submission compounds) and for in-house databases. This feature will also help with a more reliable estimation of the potential to reduce animal experiments.

#### 1.6.5 Retrieval of supporting Information

Linking (Q)SAR predictions to external data sources has found little attention in the (Q)SAR community. It is however essential for the critical evaluation of predictions and for the understanding of toxicological mechanisms. Again the problem is less trivial as it seems at a first glance and requires similar techniques as those for database aggregation. The development of new text mining techniques is crucial for the retrieval of factual information from publications.

#### 1.6.6 Interfaces

Model developers will benefit from a set of APIs that allow an easy integration, testing and validation of new algorithms. New techniques can be easily tested with relevant real-world toxicity data and compared to the performance of benchmark algorithms.

#### 1.6.7 Toxicity databases

OpenTox database work aims to integrate and provide high-quality toxicity data for predictive toxicology model development and validation. OpenTox supports the creation of dictionaries and ontologies that describe the relations between chemical and toxicological data and experiments and for the retrieval and quality assurance of toxicological information. This includes tools for chemical syntax checking, structure consolidation, and the identification of inconsistent data that requires manual inspection.

#### 1.6.8 (Q)SAR algorithms

OpenTox provides access to (Q)SAR algorithms that derive data-based predictions and models. Predictions are visualized by an application GUI or serve as input for validation routines. The open architecture is designed to allow an easy integration of external programs (open source and closed source) into any specific application.

OpenTox is starting with the integration of cheminformatics, statistical and data mining tools including functionality from other open source projects (e.g. R, WEKA [18], Taverna [19], CDK, OpenBabel [20]). A flexible plug-in architecture for applying, testing and validating algorithms interactively and systematically is used. OpenTox algorithms offer support for common tasks, such as feature generation and selection, aggregation, and visualization. The open source plug-in architecture should encourage researchers from other areas (e.g., data mining or machine learning) to integrate their methods in a safe testing environment with relevant datasets. OpenTox currently implements:

1. Algorithms for the generation and selection of features for the representation of chemicals (structure-based features, chemical and biological properties);

2. Classification and regression algorithms for the creation of (Q)SAR models;

3. Services for the combination of predictions from multiple algorithms and endpoints; and

4. General purpose algorithms (e.g. for the determination of chemical similarities, estimation of applicability domains, categorization, read across and sub-structure based database queries).

## 2. Results

### 2.1 User Requirements

User requirements indicate that we will need to provide a great flexibility with the OpenTox Framework to meet individual needs in specific applications.

*A summary of user requirements for several different kinds of OpenTox user are described in *Additional File 2.

#### 2.1.1 Use Cases

OpenTox pursues a use case driven development and testing approach. Use case development involves input from both users and developers, an internal and external peer review process, and testing approach based on user evaluation of the applications developed for the use case. Once use cases are reviewed and accepted, they are published publically on the OpenTox website.

OpenTox use cases are classified hierarchically into three classes:

Class 1: Collaboration/Project Level (e.g., 3 month development project);

Class 2: Application Level, e.g., carry out a REACH-compliant risk assessment for a group of chemicals;

Class 3: Task Level, e.g., given an endpoint (and a data set for a chemical structure category for that endpoint) develop and store a predictive model resource for a chemical space.

OpenTox Use Cases are documented by a standardised OpenTox Use Case Template describing the task, inputs, outputs, exceptions, triggers, and process resources required for the overall process and each activity step in the process. Table 1 provides an example overall process template for predicting an endpoint for a chemical structure, which the ToxPredict application described later on is based on. The user is typically a non-computational expert but knows the structure of a compound or has a chemical id or electronic structure (e.g. MOL) file. The user enters a structure via their web browser via one of three optional methods: file, paste, or sketch structure, selects the specific endpoints of interest, and starts the calculation. When the calculation is finished a report is returned.

**Table 1:** Overall Use Case process template for predicting an endpoint for a chemical structure

Activity Name:	Overall Use Case - Given a chemical structure, predict endpoints.
**Trigger Event:**	User needs toxicity prediction for one compound and initiates service request.

**Knowledge Needed (Source):**	Assume user has at least basic toxicity and chemistry knowledge but is not an expert QSAR user.

**Input Information needed (Source):**	2D Chemical Structure, toxicity endpoint(s).

**Resources needed (including services):**	Computer interface for user entry of structure, selection of endpoints and return of results. OpenTox Data Resources, Prediction Model Building and Report Generation.

**Exception Events:**	Incorrect chemical structure. Endpoint unavailable. Unable to predict endpoint.

**Knowledge Delivered (destination):**	In case of exception events direct user to further consulting and advice services.

**Output Information delivered (destination):**	Report on endpoint predictions.

**Subsequent events triggered: (relation with next activity)**	Suggestion of further Use Cases when applicable.

**Services Involved (role)**	OpenTox API, Data Resources, Prediction Model Building, Validation and Report Generation.

**Figure 1:**
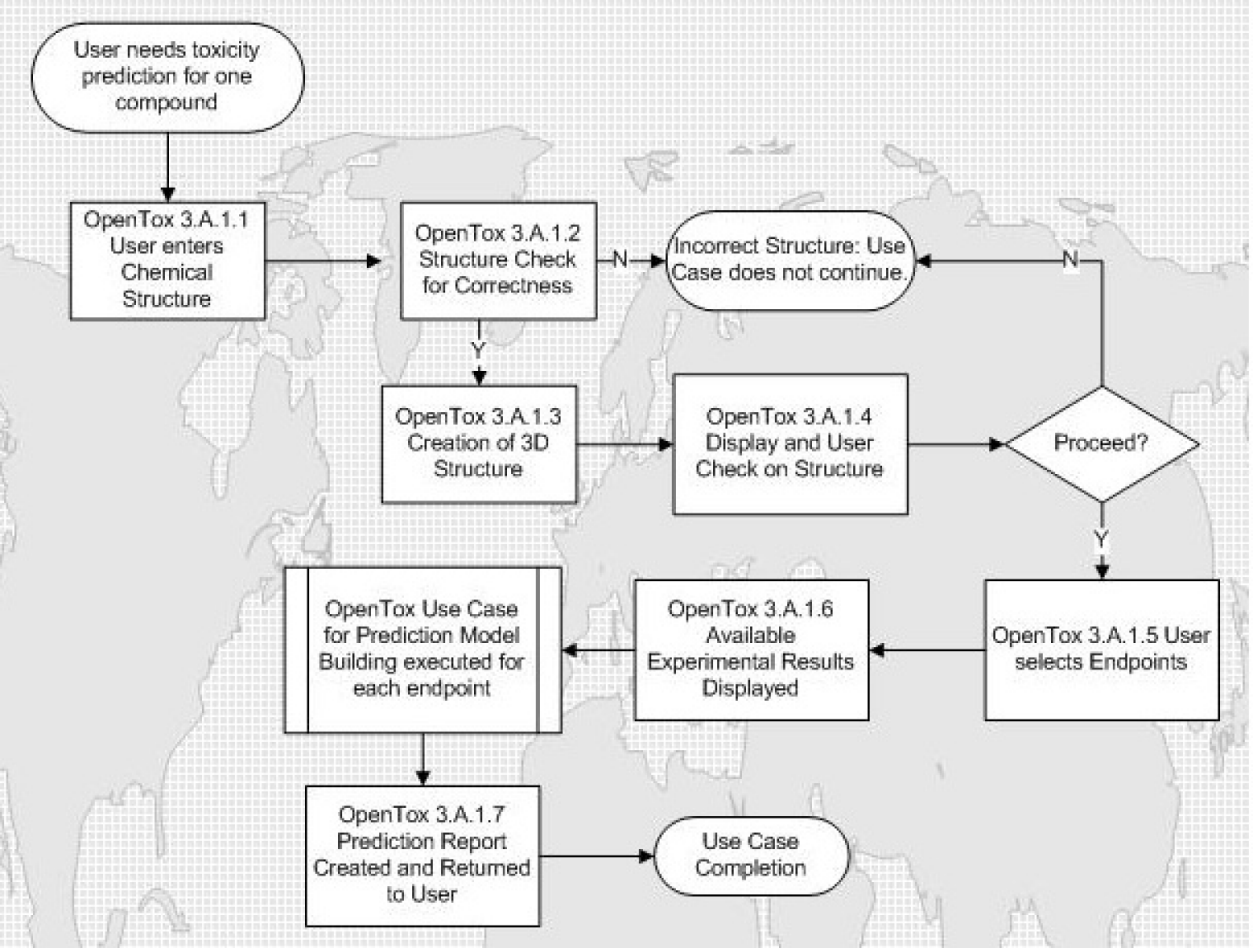
**Workflow for Use Case for predicting an endpoint for a chemical structure**.

The workflow is described in Figure 1 as the following series of steps:

1) OpenTox data infrastructure is searched for chemical id or structure;

2) The structure is checked for chemical correctness, and number of molecules;

3) Clean-up: if 2D, the structure is converted to 3D, valences saturated with hydrogen atoms, and partially optimized with molecular mechanics;

4) A check on the chemical correctness is made (bond distances, charges, valences, etc.);

5) An image of the molecule is displayed, with the results of structure check and clean-up. If serious problems with the structure are found, the user is asked if they want to continue, or if appropriate, the process is terminated automatically with an error message;

6) If experimental results for the molecule are found in the database, then the following is printed "Experimental data for this structure is available in the OpenTox database and is summarized here:";

7) All necessary descriptors are calculated, results of regression obtained, and chemical similarity to calibration molecules evaluated;

8) The prediction report is provided including the details of the basis for model prediction and including statistical reporting on the reliability of the prediction.

### 2.2 The OpenTox Framework Design

OpenTox is a platform-independent collection of components that interact via well-defined interfaces. The preferred form of communication between components is through web services. A set of minimum required functionalities for OpenTox components of various categories (prediction, descriptor calculation, data access, validation, report generation) are available on the OpenTox website [21].

OpenTox tries to follow the best practices of open source project management for core framework components. This means that source code, technical discussions and documents are open to the general public and interested parties can participate in development if they have registered for access to the developers' area of the website [22].

OpenTox is committed to the support and further development of Open Standards and Ontologies. Appendix 1 summarises some of the most important standards of relevance to the Framework.

#### 2.2.1 Architecture

OpenTox is a framework for the integration of algorithms for predicting chemical toxicity and provides:

• components for specialized tasks (e.g. database lookups, descriptor calculation, classification, regression, report generation) that communicate through well-defined language independent interfaces;

• applications that implement the capabilities of OpenTox components for specific Use Cases.

The OpenTox Framework supports building multiple applications, as well as providing components for third party applications. The Framework guarantees the portability of components by enforcing language-independent interfaces. Implementation of an integration component in a specific language/platform automatically ports the entire OpenTox Framework to that language/platform.

The OpenTox Framework is composed of:

• Components - every component encapsulates a set of functionalities and exposes them via well defined language-independent interfaces (protocols);

• Data Infrastructure adhering to interoperable principles and standards;

• Ontologies and associated services;

• Documentation and guidance for application development and use.

An OpenTox-based application implements a specific Use Case, with the appropriate user interfaces, and adhering to guidance on APIs and standards.

#### 2.2.2 Components

OpenTox components are described by templates providing documentation including minimum requirements and dependency tracking on the OpenTox website [22]. The current (Q)SAR-related component categories include Prediction, Descriptor Calculation, Data Access, Report Generation, Validation and Integration. Initial components include Rumble, Toxmatch, Toxtree, iSar, lazar, AMBIT, FreeTreeMiner, LibFminer, gSpan', MakeMNA, MakeQNA, and MakeSCR.

The interactions between components are determined by their intended use and can differ across different Use Cases, which consist of a series of steps, each applying component functionalities on input data. The interaction between components is implemented as a component. Interaction components such as workflows (e.g., Taverna) combine multiple services to offer the following functionalities:

• load the series of steps, corresponding to the specific Use Case (from a configuration file on a file system or on a network);

• take care of loading necessary components;

• execute the steps.

#### 2.2.3 OpenTox Application Programming Interfaces

To assure reliable interoperability between the various OpenTox web services, a well-defined API is required. The OpenTox APIs specify how each OpenTox web service can be used, and how the returned resources look like. It further specifies the HTML status codes returned in case of succeeded operations as well as errors codes. OpenTox interfaces have the minimum required functionalities shown in Appendix 2. The initial specifications for the OpenTox APIs have been defined and are available on the OpenTox website [23]. The initial objects already specified are Endpoint, Structure, Structure Identifiers, Feature Definition, Feature, Feature Service, Reference, Algorithm, Algorithm Type, Model, Dataset, Validation Result, Applicability Domain, Feature Selection, and Reporting.

All current OpenTox web services adhere to the REpresentational State Transfer (REST) web service architecture [24] for sharing data and functionality among loosely-coupled, distributed heterogeneous systems.

*Further information on interfaces and the REST approach is included in *Additional File 3.

The choice of employing web services allows the complete framework to operate in different locations, independent of operating systems and underlying implementation details.

**Figure 2:**
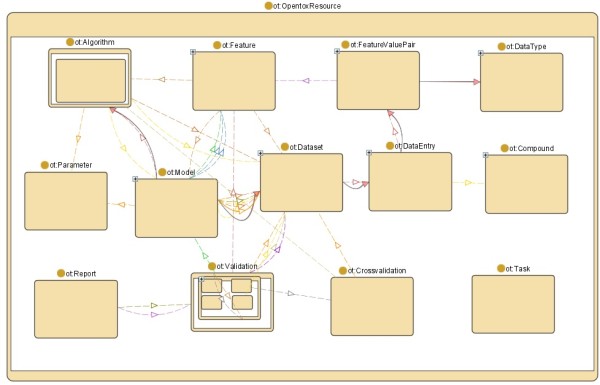
**Relationships between OpenTox Resources modelled in the OpenTox Ontology**.

Figure 2 shows the OpenTox resources modelled in the OpenTox Ontology. These resources are provided by the various OpenTox web services. The links between the components reflects interaction between the respective web services.

The model web service provides access to (prediction) models. Models are created via the algorithm web service, which supports different types of algorithms (e.g. supervised learning, feature selection, descriptor calculation, and data cleanup). Building a model will normally require various parameters, one or several datasets, as well as a set of features.

Datasets are stored in the dataset web service. A dataset contains data entries, which are chemical compounds, as well as their feature values. Features are defined as objects representing a property of a compound, including descriptors and calculated features, endpoints, and predictions. Different representations for chemical compounds can be accessed from the compound web service. The feature web service provides the available features (e.g. structural features, chemical descriptors, endpoints).

The validation web service evaluates and compares the performance of prediction models. Simple training-test-set-validation is supported as well as cross-validation. The validation result contains quality statistical figures and reports (available in html or pdf formats) that visualize the validation results. The task web service supports long-running, asynchronous processes. The ontology web service provides meta information from relevant ontologies (which can be accessed using SPARQL queries [25]), as well as lists of available services. Approaches to Authentication and Authorization will be specified in the next version 1.2 of the API.

All OpenTox resources have representations providing information about the type of resource, and what the service accepts as input such as tuning parameters. Most algorithms and model resources in OpenTox are available in multiple representations. The Resource Description Framework (RDF) representation [26], and in particular its XML formatted variant, was chosen as the master data exchange format, due to the following reasons:

• RDF is a W3C recommendation: RDF-related representations such as rdf/xml and rdf/turtle are W3C recommendations so they constitute a standard model for data exchange;

• RDF is part of Semantic Web Policy: RDF as a representation for a self-contained description of web resources contributes to the evolution of the Semantic Web; a web where all machines can "understand" each other;

• RDF is designed to be machine-readable.

Some services support additional representations like JavaScript Object Notation JSON [27], YAML [28] or Application/X-Turtle [29]. Some prediction model services provide Predictive Model Markup Language (PMML) representations [30] to improve their portability, since many machine learning applications like Weka provide support for PMML. The second version of the API, OpenTox API version 1.1, was completed and published on the OpenTox website in November 2009. Version 1.2 is scheduled for completion for September 2010 and is open to community-based input and comments on the OpenTox API pages containing more detailed information on the interfaces [23].

### 2.3 Ontologies and Controlled Vocabulary

The definition of ontology and controlled vocabulary is extremely important to the construction of the OpenTox data infrastructure. It contributes to the necessary standardization and rational organization of data, thus facilitating both vertical (e.g., within one toxicological endpoint) and horizontal (e.g., through different endpoints) retrievals. The definition consists of two main steps: first, the selection of the toxicological endpoints to be included; second, the definition of the type and extent of information for each endpoint, and their internal relationships and hierarchies.

#### 2.3.1 Schema

Two publicly available schemas for describing toxicology data are the OECD harmonised templates (OECD-HTs) [31] and the ToxML (Toxicology XML standard) schema [7]. It appears that the OECD-HTs have the advantage of being closer to the schemas established by the regulators for the industry to submit their data. However, this schema is quite generic, and does not lend easily itself to the needs of the OpenTox project in terms of scientific databases and scientific computing. On the other hand, the ToxML schema has many features necessary for accommodating large amounts of data at different levels of complexity, and for creating hierarchies within ontology constructs.

#### 2.3.2 REACH endpoints and OECD Guidelines

The OpenTox data infrastructure prioritises support of toxicological end points for which data are required under the REACH regulation. In current toxicological testing, these endpoints are addressed by both *in vitro *and animal experiments carried out according to OECD guidelines.

The toxicological endpoints considered by REACH are the following [32]: Skin irritation, Skin corrosion; Eye irritation; Dermal sensitisation; Mutagenicity; Acute oral toxicity; Acute inhalative toxicity; Acute dermal toxicity; Repeated dose toxicity (28 days); Repeated dose toxicity (90 days); Reproductive toxicity screening; Developmental toxicity; Two-generation reproductive toxicity study; Toxicokinetics; and Carcinogenicity study.

The OECD guidelines for testing of chemicals [11] are published on the Internet. Whereas there is no official list of OECD endpoints (test guidelines are developed according to the needs of member countries), and no official OECD approach to toxicity testing, interesting background information on criteria for toxicity testing has been developed as SIDS (Screening Information Data Set) [12,33,34].

#### 2.3.3 Data sources for the OpenTox data infrastructure

The main source of data for the public OpenTox data infrastructure is in the public domain, which is spread in many and varied sources and databases. They can be categorized into:

- Textual databases (e.g., IARC [35], NTP [36]);

- Machine readable files (e.g., .sdf) that include both structures and data, and that can be immediately used by modellers for (Q)SAR analyses in the OpenTox platform (e.g., DSSTox [6], ISSCAN [37], AMBIT [38], RepDose [39]);

- Large and quite complex databases on the Internet (e.g., PubChem [3], ACToR [40]).

The above differences in the types of data sources are entwined with differences in the quality of data (some databases may contain contradictory results, with no critical selection), and with changes with time (updates). Because of the varying data quality level of the various data sources, higher priority is given to databases subject to curation and quality evaluation. Databases being integrated in the first phase of OpenTox development include ISSCAN, DSSTox, CPDBAS, DBPCAN, EPAFHM, KIERBL, IRISTR, FDAMDD, ECETOC skin irritation, LLNA skin sensitisation and the Bioconcentration factor (BCF) Gold Standard Database [41,38]. Enabling access arrangements to clinical data such as that from the FDA, data from the US EPA's ToxCast [42] program, and commercial sources are also current OpenTox activities.

#### 2.3.4 OpenTox Controlled Vocabulary and Hierarchy

The OpenTox data infrastructure on toxicological data is used to support the development of (Q)SAR models within the OpenTox platform. Thus, its design takes into account the requirements of (Q)SAR modelling. A wide spectrum of (Q)SAR approaches, as applied to toxicity, exists today, ranging from coarse-grained to fine-tuned ones. Broad classes are [43]:

- structural alerts, which are substructures and reactive groups linked to the induction of chemical toxicity (e.g., carcinogenicity). They are used for preliminary hazard characterization, are quite popular with regulators and industry, and most often are based on, and provide to the users mechanistic information;

- QSARs for noncongeneric sets of chemicals (e.g., lazar, PASS [44]), which generate probabilities of being active/inactive (and to what extent) for compounds with very different structures;

- QSARs for congeneric sets of chemicals (e.g., Hansch approach), which use mechanistically-based descriptors, and describe how relatively small changes in structure can provoke variations in activity. Series of very similar (highly congeneric) chemicals are usually developed by industry.

Despite their differences, all the various (Q)SAR modelling approaches share the need of a highly structured information as a starting point. This includes the selection of ontologies, with controlled vocabulary and hierarchies.

We believe that such ontology work should be part of a public global community resource, subject to review and curation. We have created OpenToxipedia as a collaborative resource for the entry and editing of toxicology terms, supported by a Semantic Media Wiki [45]. An OpenTox Ontology Working Group is dedicated to the development and incorporation of ontologies which are relevant to OpenTox Use Cases; collaborative work on projects is supported by a Collaborative Protégé Editor. The approach is also to work with other groups with existing ontology developments so as to maximise reuse and interoperability between public ontologies.

*The OECD-HT and ToxML schema and data resource mapping experiments for the OpenTox context are described in *Additional File 4.

Based on our evaluation, we decided to adopt ToxML as the schema for data management and integration within OpenTox, and to support conversion and export to the OECD-HTs for reporting purposes.

### 2.4 Algorithms

The first tasks related to algorithms in OpenTox were to document, evaluate and discuss available and possibly interesting or useful algorithms. To make this selection more objective, we had to agree on a set of selection criteria for inclusion of algorithms in the initial OpenTox Framework development. Ongoing scientific efforts in various complementing fields have led to a high number of algorithms that are available and potentially useful for (Q)SAR and related tasks. To meet the specific user requirements and long term goals of OpenTox, it was crucial to establish a set of selection criteria.

#### 2.4.1 Algorithm Templates

To make a reasonable comparison of the available (Q)SAR algorithms possible, they were grouped into three categories: *(i) descriptor calculation algorithms, (ii) classification and regression algorithms and (iii) feature selection algorithms *(Two additional categories for clustering and consensus modelling are currently being added.). For each algorithm a short text description and a uniform (for each of the three categories) table was generated to facilitate a comparison with respect to the selection criteria. The text description of the algorithm gives a brief overview of the algorithm's background, its capabilities, dependencies and technical features. The uniform tables have three logical parts. The first one enables a black-box point of view of the algorithm and has the same fields for every algorithm category. It contains a field for the name, the input and output (semantically), the input and output format, user-specific parameters and reporting information. The second logical part is variable for the three algorithm categories and describes some intrinsic properties of the algorithms. It comprises fields for the algorithm's background and its performance. The descriptor calculation algorithms have a special field for the type of descriptor that is generated. The classification and regression algorithms have additional fields for the applicability domain and the confidence in the prediction, the bias, the type of learning (lazy or eager learning) and the interpretability of the generated model. The feature selection algorithms have special fields for type of feature selection (class-blind or class-sensitive), for the distinction of optimal, greedy or randomized methods and for the distinction of filter and wrapper approaches. The third part of the description table is again identical for the different algorithm categories. It gives information about the algorithm's availability within OpenTox, the license and dependencies, the convenience of integration, the priority of integration, the author of the algorithm and the author of the description. Additionally there are fields for a contact address (email) and for comments. Algorithm descriptions according to the template format are located on the OpenTox website [46].

*The fields of the OpenTox description table for the Algorithm Template are described in *Additional File 5.

*The initial implemented OpenTox algorithms are described in *Additional File 6.

#### 2.4.2 Algorithm Ontology

**Figure 3:**
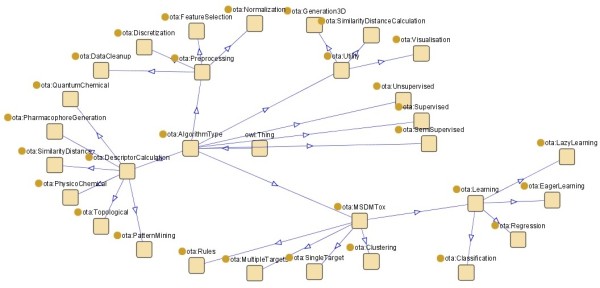
**OpenTox Algorithm Type Ontology**.

A graphical overview of the current OpenTox Algorithm ontology is shown in Figure 3.

A formal OWL [47] representation of the algorithm ontology is available on the OpenTox website [48]. The plan is to extend this ontology in the future to a full description of every algorithm, including references, parameters and default values. This will be achieved by adopting the Blue Obelisk ontology [49] and is currently work-in-progress. The RDF representation of an Algorithm contains metadata described by the Dublin Core Specifications [50] for modelling metadata (DC Namespace) and the OpenTox namespace. The establishment of an ontological base for the services facilitates the extension of the services and the introduction of new algorithms and new algorithm classes.

### 2.5 Validation

OpenTox provides unified and objective validation routines for model and algorithm developers and for external (Q)SAR programs. It implements state-of-the art procedures for validation with artificial test sets (e.g. n-fold cross-validation, leave-one-out, simple training/test set splits) and external test sets. These validation techniques are available for all (Q)SAR models (OpenTox and external programs) that are plugged into the Framework. This will help to compare algorithms and (Q)SAR models objectively and to speed up the development cycle.

#### 2.5.1 OECD Guidelines for (Q)SAR Validation

The OECD Guidelines for (Q)SAR Validation [10] addressed are as follows:

##### PRINCIPLE 1: "DEFINED ENDPOINT"

OpenTox addresses this principle by providing a unified source of well-defined and documented toxicity data. (Q)SAR model quality crucially depends on the clarity of endpoints and experimental protocols used and the ability to communicate this information in an unambiguous way, both in model development and model application. The current practice usually includes a textual description of the materials and methods used for acquiring experimental data as well as literature references, while the model description is a separate entity. The challenge to the distributed web services framework, was to provide an automatic and unique way of describing and linking the endpoint information in a formal way, able to be processed automatically by the software, with minimal human interaction. This is currently solved by making use of a simple ontology of endpoints. We have defined an ontology based on the OWL (Web Ontology Language) [47] for toxicological endpoints which is in line with current ECHA REACH guidance [51]. Using this ontology, each attribute in a toxicological dataset can be associated with an entry to the ontology, therefore allowing a unique mapping between endpoints in various and heterogeneous datasets. This ontology possesses 5 subclasses: ecotoxic effects, environmental fate parameters, human health effects, physico-chemical effects, and toxicokinetics. Each of these subclasses has one or two further layers of subclasses.

##### PRINCIPLE 2: "AN UNAMBIGUOUS ALGORITHM"

OpenTox provides unified access to documented models and algorithms as well as to the source code of their implementation. Currently OpenTox is deploying Algorithm Template descriptions and an algorithm type ontology which allows a clear definition of what type of algorithm(s) is used to construct a model.

##### PRINCIPLE 3: "DEFINED APPLICABILITY DOMAIN"

OpenTox integrates tools for the determination of applicability domains (ADs) and the consideration of ADs during the validation of (Q)SAR models. Evaluation of ADs are supported by an OpenTox algorithm API supporting situations where the AD is calculated both for situations where it is included as part of the model building application and those where it is carried out separately [52]. A specific AD algorithm is applied to a dataset, and the result is then an AD model. This model can then be applied to reason about the applicability of a model when applied to a new compound query.

##### PRINCIPLE 4: "APPROPRIATE MEASURES OF GOODNESS-OF-FIT, ROBUSTENESS AND PREDICTIVITY"

OpenTox provides scientifically sound validation routines for the determination of these measures. Within the validation part of the prototype framework, we have concentrated so far on including validation and cross-validation objects. These include a set of measures for evaluating the quality of models generated by algorithms on the datasets as summarised in Table 2.

**Table 2:** Measures for evaluating the Quality of OpenTox Models

Measures for Classification Tasks	
**Name**	**Explanation**
Confusion Matrix	A confusion matrix is a matrix, where each row of the matrix represents the instances in a predicted class, while each column represents the instances in an actual class. One benefit of a confusion matrix is that it is easy to see if the system is confusing two or more classes.
Absolute number and percentage of unpredicted compounds	Some compounds might fall outside the applicability domain of the algorithm or model. These numbers provide an overview on the applicability domain fit for the compound set requiring prediction.
Precision, recall, and F2-measure	These three measures give an overview on how pure and how sensitive the model is. The F2-measure combines the other two measures.
ROC curve plot and AUC	A receiver operating characteristic (ROC) curve is a graphical plot of the true-positive rate against the false-positive rate as its discrimination threshold is varied. This gives a good understanding of how well a model is performing. As a summarisation performance scalar metric, the area under curve (AUC) is calculated from the ROC curve. A perfect model would have area 1.0, while a random one would have area 0.5.

**Measures for Regression Tasks**	

**Name**	**Explanation**
MSE and RMSE	The mean square error (MSE) and root mean squared error (RMSE) of a regression model are popular ways to quantify the difference between the predictor and the true value.
R^2^	The explained variance (R²) provides a measure of how well future outcomes are likely to be predicted by the model. It compares the explained variance (variance of the model's predictions) with the total variance (of the data).

##### PRINCIPLE 5: "A MECHANISTIC INTERPRETATION, IF POSSIBLE"

As mechanistic interpretation often relies on human knowledge, this usually cannot be done automatically. However, in the current API it is foreseen to generate skeletons for reporting using the validation results created by extensive testing during model construction, allowing subsequent user-entered explanations about mechanisms. Other potential future extensions of OpenTox services could include resources providing insight on mechanisms, e.g. from pathways and systems biology models, selection and inclusion of *in vitro *assays relevant to the mechanism in the model, or from data mining of human adverse events data. QMRF reporting is being facilitated by the current integration of the existing QMRF editor [53] into OpenTox, this allowing end-users to annotate models with the information required by the QMRF format.

#### 2.5.2 OpenTox Approach to Validation

To guarantee a fair comparison to other algorithms, the following principles are followed:

• Separation of validation as an independent service to algorithm and model building services;

• Ability to reproduce the computational experiment (even in non-deterministic models e.g., by storing initial random values/random seeds);

• Retrieval of the exact same training and test data that was used, so that all algorithms have to work with the same data (store random seed for cross-validation);

• Use of an external validation comparison and test set that performs the same operations for all algorithms (and prevents unintended cheating).

Validation testing results are stored for subsequent retrieval because this allows obtaining information about the performance of various algorithms/models (on particular datasets) without repeating (time-consuming) experiments. This is especially useful when developing new algorithms or new versions of algorithms to allow a quick comparison to other methods.

*Three example Validation Use Cases are described in *Additional File 7.

#### 2.5.3 Validation Interfaces and Services

A Validation API is included in the OpenTox APIs ensuring the seamless interaction between all OpenTox components with regards to validation needs. Each validation resource for example, contains information about the dataset and the model, so the underlying procedures can be invoked.

*The REST service implementation for validation is described in *Additional File 8.

Further detailed information about the validation API including the approach for cross-validation can be found at http://www.opentox.org/dev/apis/api-1.1/Validation.

#### 2.5.4 Validation Application Example: Building and Validating a Model

The application example of building and validating a model is executed using the Validation web service prototype [54] (developed at the Albert Ludwigs Freiburg University (ALU-FR)) along with the lazar and fminer algorithms [13,14] (provided by In Silico Toxicology (IST)). The application is compliant with the OpenTox API, and based on interoperability between two OpenTox web services, located at two different locations: ALU-FR's services [55] and the web services of IST [56].

**Figure 4:**
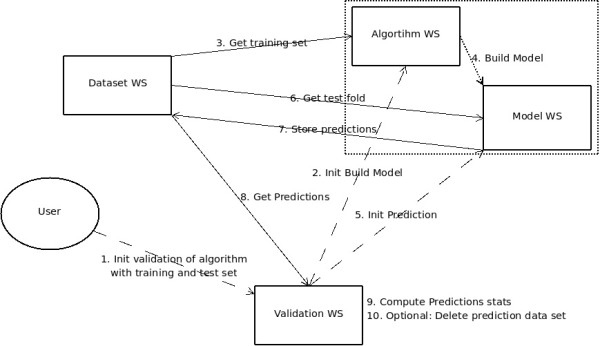
**Workflow diagram illustrating the training test set validation of a prediction algorithm**.

The goal of this Use Case is to evaluate a prediction algorithm: the algorithm trains a model on a training dataset, and then predicts the compounds of a test dataset towards a certain toxicology endpoint. The validation result reflects how well the model performed. The workflow for the training test set validation is illustrated in Figure 4. Web services are displayed as rectangles; the three key POST REST operations are symbolized as dashed lines, while solid lines visualize data flow operations.

*A description of the step by step execution of the Model Validation Use Case by the OpenTox web services is provided in *Additional File 9.

### 2.6 Reporting

The OpenTox report generating component generates reports to present the results (of predictions/model validation) to the user in a structured reporting format.

Reporting formats are guided by standards and templates such as QMRF and REACH CSR and OECD validation principles [10], which specify that to facilitate the consideration of a (Q)SAR model for regulatory purposes, it needs to be associated with the OECD Guidelines for (Q)SAR Validation.

*A description of information to be included in OpenTox reports is provided in *Additional File 10.

The different type of OpenTox reports are summarized in Table 3.

**Table 3:** Summary of Different Types of OpenTox Reports

Standard reports	
**Report type**	**Specific information included in the report**
Prediction of a single (unseen) component	Activity, applicability domain, confidence
Prediction of multiple (unseen) components	Ranking according to activity/confidence
Validation of a model	Different performance criteria (on various datasets), based on cross-validation/external test set validation
Making predictions on a particular dataset	Prediction results of various algorithms
Comparison of different models/algorithms	Ranking according to different performance criteria

**Extended reports**	

**Report type**	**Specific information included in the report**
Evaluation of a feature generation algorithm	Performance of various algorithms using the generated features compared to other features
Evaluation of a feature selection algorithm	Performance of various algorithms using the selected features compared to no feature selection

*Reporting types supported by OpenTox and the corresponding API are described in *Additional File 11.

### 2.7 OpenTox Data Infrastructure

A major pre-requisite for the successful implementation of the main principles of the Three Rs Declaration of Bologna [57] is the universal access to high quality experimental data on various chemical properties. In particular, the range of replacement alternatives methods includes the following OpenTox-relevant approaches:

• The improved storage, exchange and use of information from animal experiments already carried out, so that unnecessary repetition can be avoided;

• The use of physical and chemical techniques, and of predictions based on the physical and chemical properties of molecules;

• The use of mathematical and computer modelling, including modelling of structure-activity relationships, molecular modelling and the use of computer graphics, and modelling of biochemical, pharmacological, physiological, toxicological and behavioural processes.

Since it is likely that, in many circumstances, an animal test cannot be currently replaced by a single replacement alternative method, the development, evaluation and optimisation of stepwise testing strategies and integrated testing schemes should be encouraged. The OpenTox data facilities, made publically accessible through a web services framework, provide a solid basis for addressing the above mentioned replacement alternative goals in a more efficient, technically sound and integrated way compared to current uncoordinated practices and fragmented resources. Unfortunately, even today, more than half a century after Russell and Burchs's original publication [58] and more than 10 years after the adoption of the Three Rs Declaration of Bologna, the "state-of-the-art" is characterised by highly fragmented and unconnected life sciences data (both from a physical and ontological perspective), which is furthermore frequently inaccurate and/or difficult or even impossible to find or access. The OpenTox approach to data resource management and integration has the following major features, which address the replacement alternatives challenge and associated user, industry and regulatory needs including REACH:

• Universal database structure design, allowing for storage of multi-faceted life sciences data;

• An ontology allowing for efficient mapping of similar and/or complementary data coming from different datasets into a unifying structure having a shared terminology and meaning;

• Integration of multiple datasets with proven high-quality physico-chemical and/or experimental toxicity data;

• Built-in heuristics for automatic discovery of 2D chemical structure inconsistencies;

• Extensive support for structure-, substructure- and similarity-based searching of chemical structures;

• An OpenTox standards-compliant dataset interface that allows query submission and results retrieval from any OpenTox standards-compliant web service;

• Transparent access to and use of life sciences data, hosted at various physical locations and incorporating a variety of distributed software resources, through the OpenTox Framework.

The OpenTox initial data infrastructure includes ECHA's list of pre-registered substances [59] along with high-quality data from consortium members (e.g. ISS ISSCAN [37], IDEA AMBIT [38]), JRC PRS [60], EPA DSSTox [6], ECETOC skin irritation [61], LLNA skin sensitization [62], and the Bioconcentration Factor (BCF) Gold Standard Database [41]). Additional data for chemical structures has been collected from various public sources (e.g. Chemical Identifier Resolver [63], ChemIDplus [64], PubChem [3]) and further checked manually by experts. The database provides means to identify the origin of the data, i.e., the specific inventory a compound originated from. The data is currently publicly available and accessible via an initial implementation of the OpenTox REST data services [65], as defined in the OpenTox Framework design and its implementations.

*The *Additional File 12* on OpenTox Data Infrastructure describes in more detail the current OpenTox data facilities and resources*.

### 2.8 OpenTox Applications

We describe here the implementation of two Use Cases as applications based on the OpenTox Framework. The first case, ToxPredict, is aimed at the user having no or little experience in QSAR predictions. This Use Case should offer an easy-to-use user interface, allowing the user to enter a chemical structure and to obtain in return a toxicity prediction for one or more endpoints. The second case, ToxCreate, is aimed at the experienced user, allowing them to construct and to validate models using a number of datasets and algorithms.

Both Use Cases also demonstrate inter-connectivity between multiple OpenTox services. Within ToxPredict, web services from three different service providers (TUM, IDEA, and NTUA) are operating together. In ToxCreate the model construction is performed using IST web services, while the validation and reporting is executed using ALU-FR services.

#### 2.8.1 ToxPredict Application

As the ToxPredict Use Case should offer easy access to estimate the toxicological hazard of a chemical structure for non-QSAR specialists, one main aim was to design a simple yet easy-to-use user interface. For this, one of the goals was also to reduce the number of possible parameters the user has to enter when querying the service. The Use Case can be divided into the following five steps:

1. Enter/select a chemical compound

2. Display selected/found structures

3. Select models

4. Perform the estimation

5. Display the results

The ToxPredict graphical user interface is shown in Figure 5; the interaction and sequence of OpenTox services interoperating during the different steps of the ToxPredict application execution are detailed in Figures 6, 7, 8, 9, 10, 11 and 12.

**Figure 5:**
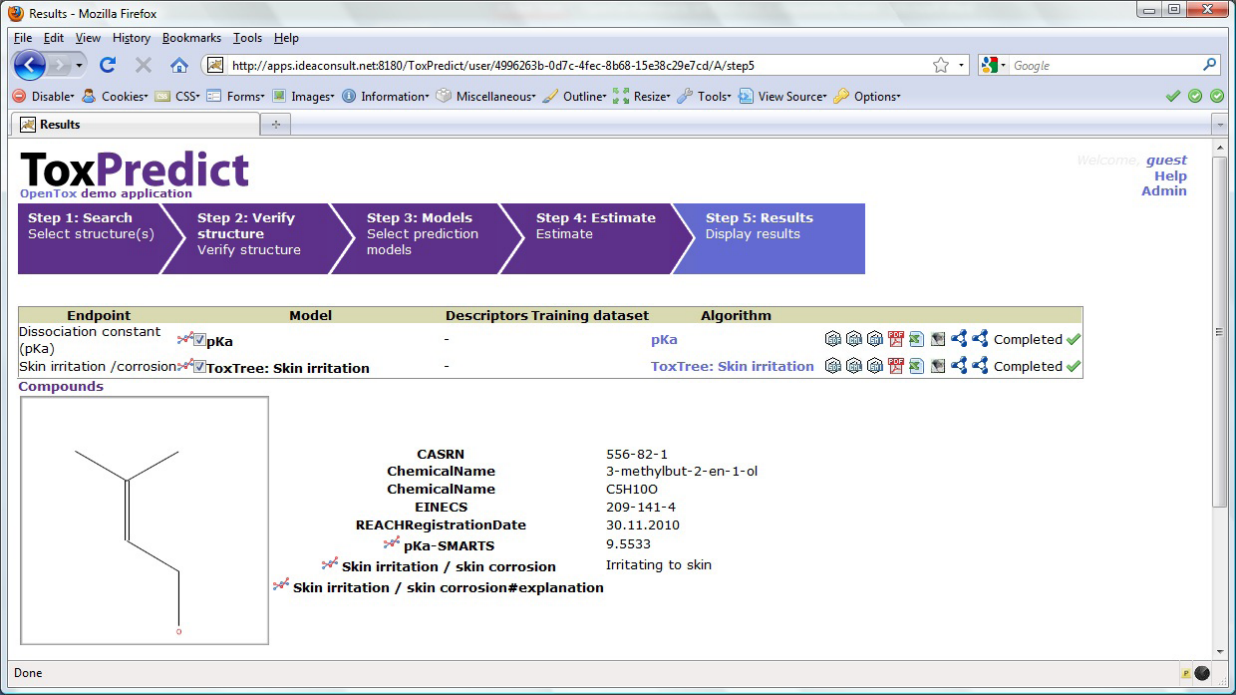
**Display of results from Step 5 of ToxPredict Application**.

**Figure 6:**
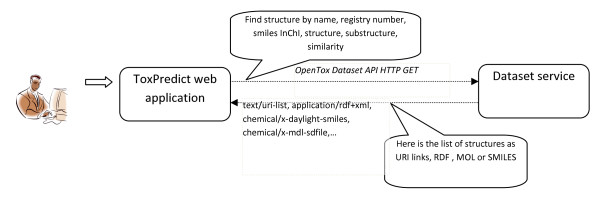
**ToxPredict Step 1 - Enter Compound, Interaction of OpenTox Services**.

**Figure 7:**
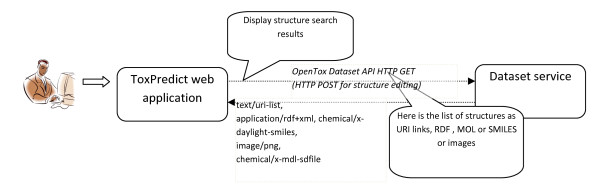
**ToxPredict Step 2 - Structure Selection, Interaction of OpenTox Services**.

**Figure 8:**
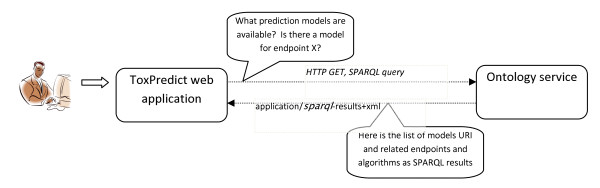
**ToxPredict Step 3 - Model Selection, Interaction of OpenTox Services: User-System Interaction**.

**Figure 9:**
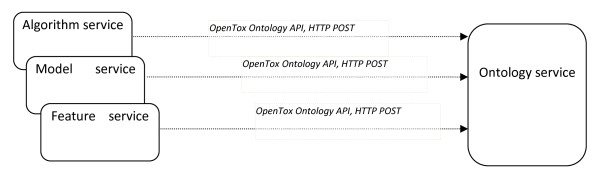
**ToxPredict Step 3 - Behind the scenes: previously, algorithm, model and feature services had registered a list of algorithms, models and features into the Ontology service, by POSTing the URIs of these objects**.

**Figure 10:**
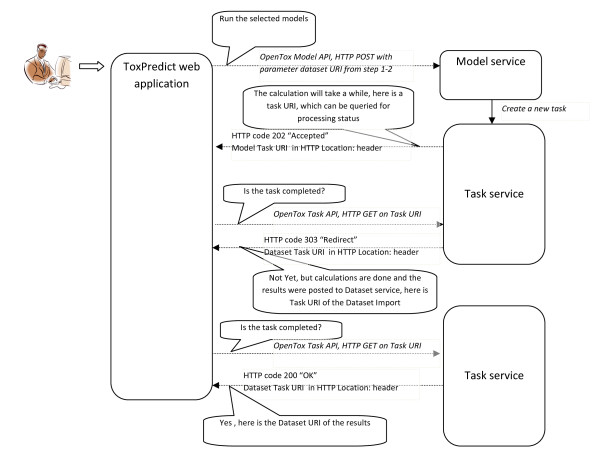
**ToxPredict Step 4 - Model Estimation, Interaction of OpenTox Services: User-System Interaction**.

**Figure 11:**
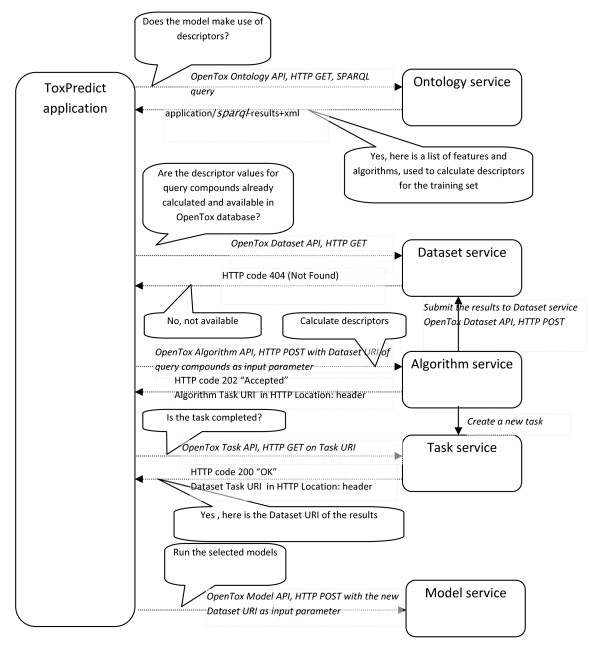
**ToxPredict Step 4 - Model Estimation, Interaction of OpenTox Services: Behind the scenes**.

**Figure 12:**
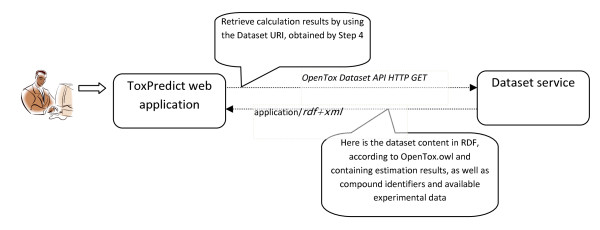
**ToxPredict Step 5 - Display Results, Interaction of OpenTox Services**.

*A detailed step-by-step graphical interface description of the ToxPredict workflow steps are provided in *Additional File 13.

The following sequence of descriptions explains the workflow and operations of the example ToxPredict user session.

##### ToxPredict Step 1 - Enter/select a chemical compound

The first step in the ToxPredict workflow provides the means to specify the chemical structure(s) for further estimation of toxicological properties. Free text searching allows the user to find chemical compounds by chemical names and identifiers, SMILES [66] and InChI strings, and any keywords available in the OpenTox data infrastructure. The data infrastructure contains information from multiple sources, including the ECHA pre-registration list.

##### ToxPredict Step 2 - Display selected/found structures

The second step displays the chemical compounds, selected by the previous step. The user interface supports the selection/de-selection of structures, and editing of the structures and associated relevant information. The OpenTox REST Dataset services are used in this step of the application in order to retrieve the requested information.

##### ToxPredict Step 3 - Select models

In the third step, a list of available models is displayed. Links to training datasets, algorithms and descriptor calculation REST services are provided. The models provide information about the independent variables used, the target variables (experimental toxicity data) and predicted values. All these variables are accessible via the OpenTox Feature web service, where each feature can be associated with a specific entry from the existing endpoint ontology. The association is usually done during the upload of the training data into the database. The endpoint, associated with the model variables is automatically retrieved and displayed in the first column of the list. This provides an automatic and consistent way of complying with the first OECD validation principle of using a "Defined endpoint".

This step involves an interplay between multiple OpenTox web services. Algorithm, Model, and Feature services are registered into the Ontology service, which provides RDF triple storage with SPARQL, allowing various queries. The ToxPredict application queries the Ontology service for all available models, along with the associated information about algorithms used in the model, descriptors, and endpoints. The list of models may include models, provided by different partners and running on several remote sites (TUM and IDEA models are shown in this example). The Ontology service serves like a hub for gathering a list of available models and algorithms from remote sites. There could be multiple instances of the ToxPredict application, configured to use different Ontology services, and therefore, allowing for a different subset of models to be exposed to end users.

##### ToxPredict Step 4 - Perform the estimation

Models, selected in Step 3 are launched in Step 4, where the user can monitor the status of the processing. The processing status is retrieved via OpenTox Task services. Different Model, Algorithm, Dataset, and Ontology services, running on different remote locations can be involved at this stage. If a model relies on a set of descriptors, an automatic calculation procedure is performed, which involves launching a descriptor calculation by remote Algorithm services. The procedure is as follows:

The Ontology service is queried to retrieve information about the independent variables, used in the model. If no such variables are involved (e.g., in case of ToxTree models, which rely on chemical structure only), the workflow proceeds towards model estimation. In case of a model, based on descriptors (e.g., a regression model), the procedure is slightly more complex, as explained below.

Each independent variable is represented as a Feature and managed via the Feature service. Each feature has associated a web address (OWL property opentox:hasSource from OpenTox OWL ontology), which specifies its origin. The tag could point to an OpenTox Algorithm or Model service, in case it holds a calculated value, or point to a Dataset service, in case it contains information, uploaded as a dataset (for example experimental endpoint data). If the feature origin is a descriptor calculation algorithm, the web address points to the Algorithm service, used to calculate descriptor values, and the same web address can be used again via the OpenTox Algorithm API in order to calculate descriptors for user-specified structures. The Algorithm services perform the calculation and store results into a Dataset service, possibly at a remote location. Then finally, a dataset with all calculated descriptor values is submitted to the Model service. Upon estimation, Model results are submitted to a Dataset service, which could be at a remote location, which could be the same or different to that for the model services.

The interplay of multiple services, running on remote sites, provide a flexible means for the integration of models and descriptors, developed by different organisations and running in different environments. Identification of algorithms and models via web URIs ensure the compliance with the OECD validation principle 2 of "An unambiguous algorithm", as well as repeatability of the results of the model building. Extensive meta information about the algorithm and models themselves is accessible via web URIs and the OpenTox API.

##### ToxPredict Step 5 - Display the results

The final step displays estimation results (see Figure 5), as well as compound identification and other related data. Initial demonstration reports in several formats can be accessed via icons on the right hand side of the browser display.

ToxPredict is a demonstration web application, providing a user-friendly interface for estimating toxicological hazards. It provides a front end to multiple OpenTox services, currently integrating IDEA ontology, dataset, feature and model services with TUM descriptor calculation and model services and NTUA algorithm services. Future work will include integration of other third party model services, as well as Validation and Reporting services. While current functionality may appear to an end-user not much different from a stand-alone prediction application like ToxTree, the back-end technology provides a very flexible means for integrating datasets, models and algorithms, developed by different software technologies and organisations and running at remote locations.

#### 2.8.2 ToxCreate application

The ToxCreate Use Case, in contrast to ToxPredict, is aimed at researchers working in the life sciences and toxicology, QSAR experts, and industry and government groups supporting risk assessment, who are interested in building predictive toxicology models. It allows the creation of a number of models using one or more algorithms. Therefore it is not as easy to use as the ToxPredict application, as not only the algorithm has to be selected, but also the right parameter setting needs to be explored; these parameters are algorithm-dependent. For this decision-making, the expert has to have sound knowledge of the algorithm they are using.

The following sequence of steps explains the execution of a sample session of the ToxCreate application:

*A graphical interface description of the ToxCreate workflow steps are provided in *Additional File 14.

##### ToxCreate Step 1 - Upload Dataset

The first step of the ToxCreate workflow enables the user to specify a model training dataset in CSV format, consisting of chemical structures (SMILES) with binary class labels (e.g. active/inactive). The file is uploaded to the server and labelled with a user-defined name. In contrast to ToxPredict, users can specify their own training data/endpoint. This is done in batch mode, i.e. without interactive screens to select chemicals based on different criteria, which is convenient for expert users. By hitting "Create model", a QSAR model is derived. The current prototype demonstrates lazar models only. No model parameters can be set at this time, but future versions will enable arbitrary OpenTox API-compliant models.

##### ToxCreate Step 2- Create and Display Model

This next step in ToxCreate displays information about the model learned from the data submitted in the previous step. It features status information, date and number of compounds present in the dataset. A link leads to the complete specification of the model in OWL-DL. Through integration with the OpenTox Validation service, it is possible to validate the model and select the most appropriate models for further evaluation. At this point, the model is permanently stored on the server and can be used for predictions at any time in the future.

##### ToxCreate Step 3 - Select and Use Model(s) for Prediction

In this step, a chemical (specified via SMILES code) can be entered in order to predict its chemical behaviour by arbitrary models existing on the server (note that in this way, arbitrary combinations of model algorithms and datasets/endpoints are available to test the structure).

##### ToxCreate Step 4 - Display Prediction Results

Step 4 displays the predictions made by the selected models from the previous step along with an image of the predicted structure. Based on the selections made in the previous step, the expert user may predict the same structure by a variety of algorithms for the same dataset/endpoint and compare the predictions. Together with model validation, users are able to use ToxCreate to select appropriate models with adjusted parameters beforehand. By predicting a variety of related endpoints, instead of just one, combined with arbitrary models at the same time, ToxCreate enables free predictive toxicology modelling exploration along different dimensions.

## 3. Discussion

The OpenTox Framework supports the development of *in silico *predictive toxicology applications based on OpenTox components for data management, algorithms and validation. Initial applications are being provided openly to users and developers through the OpenTox website and linked services including partner resources. Such applications support users in the development and training of QSAR models against their own toxicological datasets, e.g., they may upload a dataset for a given endpoint to an OpenTox service, define a variety of parameters and build and download a model. Subsequent releases in 2010 and 2011 will extend the Framework to the support of a broader range of computational chemistry and biology modelling approaches, and integration of data from new *in vitro *assays, and refine the API designs based on development experiences on the effectiveness of applications in supporting integrated testing strategies as required by REACH.

OpenTox provides a platform technology with:

1. a unified interface to access toxicity data and *in silico *models;

2. a framework for the development and validation of new (Q)SAR models;

3. a framework for the development, validation and implementation of new *in silico *algorithms; and

4. well defined standards for the exchange of data, knowledge, models and algorithms.

OpenTox currently provides high-quality data and robust (Q)SAR models to explore the chronic, reproductive, carcinogenic and genotoxic toxicity of chemicals. The integration of further toxicological endpoints should be straightforward with OpenTox tools and standards.

OpenTox is tailored especially to meet the requirements of the REACH legislation and to contribute to the reduction of animal experiments for toxicity testing. It adheres and supports the *OECD Guidelines for (Q)SAR Validation *and incorporates the *QSAR Model Reporting Format (QMRF) *from the EC Joint Research Council (EC JRC). Relevant international authorities (e.g., EC JRC, ECVAM, EPA, FDA) and industry organisations participate actively in the advisory board of the OpenTox project and provide input for the continuing development of requirement definitions and standards for data, knowledge and model exchange.

OpenTox will actively support the further development and validation of *in silico *models and algorithms by improving the interoperability between individual systems (common standards for data and model exchange), increasing the reproducibility of *in silico *models (by providing a common source of structures, toxicity data and algorithms) and by providing scientifically-sound and easy-to-use validation routines. For this reason it is likely that the predictive toxicology application development cycle will speed up which will lead to improved and more reliable results. As OpenTox offers all of these features openly to developers and researchers, we expect an international impact that goes beyond a single research project. For organisations, that cannot afford a dedicated computational toxicology department, the OpenTox community provides an alternative affordable source of solutions and expertise.

Biotech and pharmaceutical industry SMEs will benefit from the OpenTox project, because it will provide access to toxicological information and *in silico *models from a single, easy-to-use interface that is publicly available. OpenTox should reduce the costs for product candidate development by providing new resources for toxicity screening at a very early stage of product development, thus eliminating toxic liabilities early and reducing the number of expensive (and sometimes animal consuming) efficacy and toxicity experiments. With the OpenTox Framework it will also be possible to identify substructures that are responsible for toxicity (or detoxification), and information that can be used for the design of safer and more efficient products.

The ECB estimated that 3.9 million additional animals could potentially be used for the initial implementation of the REACH program (A more recent evaluation based on REACH chemical pre-registrations at ECHA indicate an even larger testing requirement [67]). Chronic effects such as reproductive and developmental toxicity, *in vivo *mutagenicity and carcinogenicity will require ~72% of the test animals (~2.8 million animals). In the same study a 1/3 - 1/2 reduction potential was estimated for (Q)SAR techniques available at that time (2003). As OpenTox focuses initially on the development of improved (Q)SAR techniques for reproductive, developmental and repeated dose toxicity, and for *in vivo *mutagenicity and carcinogenicity endpoints, it could contribute substantially to an estimated reduction potential of 1.4 million animals alone for REACH. A more detailed analysis of replacement possibilities under consideration of applicability domains is being currently pursued.

The OpenTox Framework works independently of the toxicity endpoint. As it will be easy to plug in databases for other endpoints, it is likely that significant savings will occur also for other endpoints (e.g. ecotoxicity endpoints from the FP7 Environment Theme ENV.2007.3.3.1.1). An exciting opportunity in this respect is the inclusion of human data from epidemiological and clinical studies and the utilization of data from adverse effect reporting systems, because in this case no data from animal experiments will be needed.

## 4. Conclusions

This work provides a perspective on the growing significance of collaborative approaches in predictive toxicology to create the OpenTox Framework as a public standards-based interoperable platform. Key challenges to be overcome are both technical and cultural and involve progressing issues related to cross-organisational, enterprise and application interoperability, knowledge management and developing a culture and framework supporting a community-based platform and collaborative projects emerging from the community foundation [68-70]. The OpenTox Framework offers a standardized interface to state-of-the art predictive toxicology algorithms, models, datasets, validation and reporting facilities on the basis of RESTful web services and guided by the OECD Principles, REACH legislation and user requirements.

Initial OpenTox research has provided tools for the integration of data, for the generation and validation of (Q)SAR models for toxic effects, libraries for the development and integration of (Q)SAR algorithms, and scientifically-sound validation routines. OpenTox supports the development of applications for non-computational specialists in addition to interfaces for risk assessors, toxicological experts and model and algorithm developers.

The OpenTox prototype established a distributed state-of-the-art data warehousing for predictive toxicology. It enables improved storage, exchange, aggregation, quality labelling, curation and integrated use of high quality life sciences information, and allows for consistent and scientifically sound mathematical and computer modelling, including modelling of structure-activity relationships for REACH-relevant endpoints.

A key decision towards algorithm implementation was the adoption of the REST architectural style, because it is suitable for achieving three important goals: independent deployment of components, ease of standardised communication between components and generality of interfaces. These advantages will enable the development and integration of additional algorithms in the future, which may be offered by a variety of third-party developers in the community. Ongoing maintenance and addition of novel predictive algorithms relevant to predictive toxicology will contribute to the long-term sustainability of OpenTox in generating valuable resources for the user scientific community.

Many descriptor calculation algorithms and QSAR modelling methods have already been implemented and incorporated within OpenTox. These include methods provided by OpenTox partners and algorithms contained in other state-of-the-art projects such as WEKA and CDK. Descriptor calculation algorithms are able to generate both physico-chemical and sub-structural descriptors. QSAR modelling methods cover a wide range of approaches and address many user model building requirements, since they include regression and classification algorithms, eager and lazy approaches, and algorithms producing more easily interpretable and understandable models. The initial prototype also includes implementations of clustering algorithms and feature selection tools. Within OpenTox we have also implemented basic validation routines, simple validation (with supplied test set or training/test split), cross-validation routines (including leave-one-out), as well as making initial reporting routines available.

The OpenTox Framework supports rapid application development and extensibility by using well-defined ontologies, allowing simplified communication between individual components. Two user-centered prototype applications, ToxCreate and ToxPredict, show the potential impact of the framework regarding high-quality and consistent structure-activity relationship modelling of REACH relevant endpoints. The applications have been made available publically on the Web [71] providing immediate access to the applications as they have been developed. Considerable additional materials and references [72-128] have been provided with this paper to support as complete a description of OpenTox as possible for users and developers.

ToxPredict satisfies a common and important situation for a user wishing to evaluate the toxicity of a chemical structure. The user does not have to cope with many current challenges such as the difficulty of finding or using existing data or the complications of creating and using complicated computer models. Because of the extensible nature of the standardised design of the OpenTox Framework, many new datasets and models from other researchers may be easily incorporated in the future, both strengthening the value offered to the user and ensuring that research results are not left languishing unused in some isolated resource not accessible to the user. The approach offers the potential to be extended to the complete and easy-to-use generation of reporting information on all REACH-relevant endpoints based on existing available scientific research results, and indications when additional experimental work is required, thus satisfying currently unmet industry and regulatory needs.

ToxCreate provides a resource to modellers to build soundly-based predictive toxicology models, basely solely on a user-provided input toxicology dataset that can be uploaded through a web browser. The models can be built and validated in an automated and scientifically sound manner, so as to ensure that the predictive capabilities and limitations of the models can be examined and understood clearly. Models can subsequently be easily made available to other researchers and combined seamlessly into other applications through the OpenTox Framework.

Continuing effort will be carried out by OpenTox developers to meet current academic and industry challenges regarding interoperability of software components and integration of algorithm and model services within the context of tested Use Cases. The approach to interoperability and standards lays a solid foundation to extend application development within the broader developer community to establish computing capabilities that are sorely missing in the field of predictive toxicology today, and which are holding back advances in both R&D and the application of R&D project outcomes to meet industry and regulatory needs.

## 6. List of Abbreviations

AD: Applicability Domain; ALU-FR: Albert Ludwigs University Freiburg; API: Application Programming Interface; BCF: Bioconcentration Factor; CDK: Chemistry Development Kit; CPDB: Carcinogenic Potency Database; EC: European Commission; ECB: European Chemicals Bureau; ECETOC: European Centre for Ecotoxicology and Toxicology of Chemicals; ECha: European Chemicals Agency; ECVAM: European Centre for the Validation of Alternative Methods; EPA: Environmental Protection Agency; ER: Endocrine Receptor; EU: European Union; FDA: Food and Drug Administration; FHG ITEM: Fraunhofer Institute for Toxicology & Experimental Medicine; FP7: Seventh Framework; GUI: Graphical User Interface; IDEA: Ideaconsult Ltd; IUCLID5: International Uniform Chemical Information Database 5; INCHI: IUPAC International Chemical Identifier; ISS: Istituto Superiore di Sanità; ISSCAN: ISS Carcinogenicity Database; JRC: Joint Research Council; JRC: PRS JRC Pre-registered Substances; LLNA: Local Lymph Node Assay; MOA: Mechanism of Action; NTP: National Toxicology Program; NTUA: National Technical University of Athens; OECD: Organisation for Economic Co-operation and Development; OECD-HT: OECD Harmonized Templates; OWL: Web Ontology Language; PLS: Partial Least Squares; QMRF: (Q)SAR Model Reporting Format; QPRF: (Q)SAR Prediction Reporting Format; (Q)SAR: (Quantitative) Structure-Activity Relationship; RDF: Resource Description Framework; REACH: Registration, Evaluation, Authorisation and Restriction of Chemicals; REPDOSE: Repeated Dose Toxicity Database; REST: REpresentational State Transfer; SMILES: Simplified Molecular Input Line Entry Specification; SVM: Support Vector Machine; URI: Universal Resource Index; XSD: XML Schema Definition; XML: Extensible Markup Language; TOXML: Toxicology Markup Language.

## 7. Competing interests

The authors declare that they have received research funding for this work from the European Commission under its Seventh Framework Program. Otherwise the authors declare that they have no competing interests.

## 8. Authors' contributions

BH fulfilled the principal investigator role coordinating the activities of requirements analysis, research and development, and drafted the manuscript. ND provided created design components for OpenTox templates and interfaces. CH led the OpenTox Framework and API design activities and the development of the OpenTox ToxCreate application. MR carried out technical implementation of OpenTox web resources. NJ played a leadership role in OpenTox Framework and API design activities, implementation of the OpenTox data services and the development of the OpenTox ToxPredict application. VJ performed chemical data collection, analysis and curation, led OpenTox testing activities and helped to draft the manuscript. IN helped in the design of RDF representations of OpenTox API objects and provided guidance for ontology development related issues. RB participated in high quality toxicity database preparation and in the discussion of the results. OT participated in the development of ontology for toxicological endpoints. OT and RB participated in validation of available schemas for describing toxicology data. OT mapped a number of databases to the ToxML and OECD-HT schemas. SK played a leadership role in OpenTox Framework and API design activities and led the work activities on OpenTox algorithms. TG, FB and JW worked on the OpenTox API and algorithm implementation. AK worked on the OpenTox API and validation and reporting service design. MG worked on the OpenTox API and validation and reporting service implementation. AM worked on the OpenTox API and fminer descriptor calculation service implementation. HS worked on the OpenTox API and the algorithms prototype implementation. GM worked on use case development and documentation. AA worked on the application of QSAR algorithms to publicly available datasets. PS worked on the OpenTox API, the algorithms prototype implementation and use case development. PS worked on the OpenTox API and the algorithms prototype implementation. DG led the activities on graphical user interface design and specifications. VP participated in the development of controlled vocabulary and in the discussion of the results. DF worked on the OpenTox API and the algorithms prototype implementation for MakeMNA, MakeQNA, and MakeSCR. AZ worked on the MakeMNA and MakeQNA descriptor calculation service implementation. AL participated in the development of ontology for toxicological endpoints and OpenToxipedia. TG participated in the development of OpenToxipedia. SN participated in the development of the controlled vocabulary and in high quality toxicity database preparation. NS participated in the development of the controlled vocabulary. DD worked on the OpenTox API, and MakeMNA and MakeQNA descriptor calculation service implementation. SC provided customer inputs for use case development from pharma and R&D Labs. IG provided the initial concept for the MaxTox algorithm and prediction logic. SR developed the application and its API compliance for the model generation of MaxTox. HP developed the MaxTox Random Forest models in R. SE developed ontologies and use cases for repeated dose toxicity. All authors read and approved the final manuscript.

## 9. Authors' information

Barry Hardy (BH) manages the eCheminfo and InnovationWell community of practice and research activities of Douglas Connect, Switzerland. He obtained his Ph.D. in 1990 from Syracuse University working in the area of computational chemistry, biophysics and computer-aided molecular modelling and drug design. Over the past 20 years BH has led numerous international projects in the area of the chemical, life and medical sciences. He has developed technology solutions for internet-based conferencing, tutor-supported e-learning, laboratory automation systems and computational chemistry and informatics. BH was a National Research Fellow at the FDA Center for Biologics and Evaluation, a Hitchings-Elion Fellow at Oxford University and CEO of Virtual Environments International. He is currently coordinating the OpenTox FP7 project.

The owner of *in silico toxicology *Christoph Helma (CH) has received his Ph.D. in chemistry and a Masters in toxicology. His main research interest is the application of data mining techniques to solve real-world toxicological problems. He has more than 10 years experience in predictive toxicology research and has published more than 40 peer reviewed research papers. He was editor for the "Predictive Toxicology" textbook and editor for special sections in "Bioinformatics" and "Combinatorial Chemistry and High Throughput Screening", invited speaker for major (Q)SAR conferences and main organizer of the "Predictive Toxicology Challenge". CH has developed and implemented the lazar program, that was awarded with the Research Prize for Alternative Methods to Animal Experiments (German Federal Ministry on Consumer Protection, Food and Agriculture, 2005) and the Research Prize for Cancer Research without Animal Experiments (Doctors Against Animal Experiments, 2006). He is currently developing an Inductive Database for the FP6 Sens-it-iv project.

Nina Jeliazkova (NJ): M.Sc. in Computer Science, Institute for Fine Mechanics and Options, St. Petersburg, Russia - 1991, Ph.D. in Computer Science, Sofia, Bulgaria (Thesis "Novel computer methods for molecular modelling") - 2001. Research fields - data mining, cheminformatics, QSAR, networking. Professional Experience - software developer at the oil refinery Neftochim, Bourgas, Bulgaria - 1991-1995, researcher at the Central Laboratory for Parallel Processing, Bulgarian Academy of Sciences, Sofia, Bulgaria - 1996-2001, collaborator and software developer with the Laboratory of Mathematical Chemistry, Bourgas, Bulgaria - 1996-2001, PostDoc at Central Product Safety department, Procter & Gamble, Brussels, Belgium - 2002-2003, associate professor at the Institute for Parallel Processing, Bulgarian Academy of Science, Sofia, Bulgaria 2004 - now, technical manager and co-owner of Ideaconsult Ltd. - 2005 - now. Teaching - Computer Graphics, Computer architectures, Operating Systems, Internetworking at Technical University - Sofia, New Bulgarian University - Sofia, American College - Sofia, Bulgaria. Author and co-author of about 40 scientific papers in Bulgarian and international journals and textbooks. A list of selected publications is available at http://ambit.acad.bg/nina. Research interests: QSAR, applicability domain, data mining methods, network protocols. Experience in software development, QSAR, cheminformatics.

Vedrin Jeliazkov (VJ): M.Sc. in Computer Science from Université Paris 7 Diderot, Paris, France. Professional experience: software developer, responsible for the design of quality assurance tests - R&D department of Electricité de France (EDF), Clamart, France - 1996-1998; research associate and assistant professor at the Central Laboratory for Parallel Processing - Bulgarian Academy of Sciences (now Institute for Parallel Processing) - 1998-2001, 2003-2007; network engineer at the core node of the Bulgarian National Research and Education Network - 1998-2001, 2003-2007; scientific officer at the European Commission, DG Information Society, Directorate E, Essential Information Society Technologies and Infrastructures - 2001-2002; Chief Technical Officer of the Bulgarian National Research and Education Network - 2004-2007; Chief Technical Director of the Bulgarian National Supercomputing Centre - 2008, researcher at and co-owner of Ideaconsult Ltd - 2004 - present. Research interests: network monitoring and security, parallel and quantum information processing. Participated in numerous R&D projects in France, Belgium and Bulgaria, authored nine research papers, co-authored one book and gave several talks in scientific conferences.

Ivelina Nikolova (IN): M.Sc. in E-learning from University of Sofia, Bulgaria, M.Sc. in Economics from University of Sofia, Bulgaria, B.Sc. in Computer Science from University of Sofia, Bulgaria. Professional experience: software developer at Linguistic Modelling Department, Institute for Parallel Processing, Bulgarian Academy of Sciences, Sofia - 2001 - present. Research interests: Natural Language Processing (Computational Linguistics), e-Learning, Software Engineering, Quality and Reliability. Participated in numerous R&D projects, authored three research papers and participated in several scientific conferences.

Romualdo Benigni (RB) is the leading expert of the ISS for (Q)SAR. He has participated in several EU funded projects aimed at evaluating experimental mutagenicity systems from a toxicological point of view, and to projects on the evaluation of (Q)SAR models for the prediction of mutagenicity and carcinogenicity. He is the Italian representative in the EU *ad hoc *Group on (Q)SAR, and in the OECD *ad hoc *Group and Steering committee on (Q)SAR. His research activities include: Molecular biology; Environmental chemical mutagenesis; Statistics and mathematical modelling; Structure-Activity Relationships; Chemical Relational Databases. He organized and co-organized workshops/seminars/schools on (Q)SAR and modelling, including:

• "Quantitative modelling approaches for understanding and predicting mutagenicity and carcinogenicity" Rome, 3-5 September 1997.

• "Complexity in the Living: a problem-oriented approach" Rome, 28-30 September 2004.

• "(Q)SAR models for mutagens and carcinogens" Rome, 22-23 June 2006.

RB is author or co-author of about 150 papers in international journals and books. He is on the Editorial Board of the "Journal of environmental science and health, part C, Environmental Carcinogenesis and Ecotoxicology Reviews", and "Current Computer Aided Drug Design".

Dr. Olga Tcheremenskaia (OT) is a chemist, Masters Degree (1997) in biotechnology and organic synthesis from Moscow M.V. Lomonosov State Academy of Fine Chemical Technology, Ph.D. in bioorganic chemistry (2000) from the Chemical Physics Institute, Russian Academy of Sciences, Moscow. Since 2001 she is working at Istituto Superiore di Sanità (ISS), Rome, Italy. She participated in different Italian and international research projects with the following research activities: bioinformatics, proteomics, molecular characterization of viral strains, cheminformatics, toxicological and genetic database development. In 2008 OT joined the Computational Carcinogenicity Unit of the Environment and Health Department of ISS. Her research activities include: development of algorithms for mutagenicity and carcinogenicity prediction, organization of chemical databases, validation of different schemas for toxicity data integration, mapping between different toxicological databases, and the development of ontology for toxicological endpoints.

Stefan Kramer (SK) is professor of bioinformatics at the computer science department of Technische Universität München. After receiving his doctoral degree from the Vienna University of Technology, he spent a few years as an assistant professor in the Machine Learning lab of the University of Freiburg. He was the co-organizer of the Predictive Toxicology Challenge 2000-2001, an international competition in toxicity prediction. He has organized several conferences and workshops, edited special issues of journals, given invited talks and tutorials, and serves on the program committees of major data mining and machine learning conferences and on the editorial board of the Machine Learning journal. His current research interests include data mining, machine learning, and applications in chemistry, biology and medicine.

Andreas Karwath (AK) has recently become interested in the field of cheminformatics after receiving his PhD in the fields of computational biology and data-mining in 2002 from the University of Wales, Aberystwyth. His main research topics are the application of data-mining and machine learning for structured data. He has been involved in a number of applications in bio- and chem- informatics, including remote homology detection, functional class prediction of unknown genes, and the alignment of relational sequences with the REAL system. AK is the main developer of the SMIREP prediction system that is available on the Internet http://www.karwath.org/systems/smirep. The SMIREP system allows the reliable prediction of various (Q)SAR endpoints, mainly employing the SMILES code of the compounds under consideration. AK is also on the editorial board of the *The Open Applied Informatics Journal*, served as member of the program committee for a number of well-known international conferences as well as being a reviewer for journals like *JMLR*, *Bioinformatics*, *Machine Learning*, and *JAIR*.

Haralambos Sarimveis (HS) received his Diploma in Chemical Engineering from the National Technical University of Athens (NTUA) in 1990 and the M.Sc. and Ph.D. degrees in Chemical Engineering from Texas A&M University, in 1992 and 1995 respectively. Currently, he is the director of the "Unit of Process Control and Informatics" in the School of Chemical Engineering at NTUA. His main research directions are in process control and computational intelligence (neural networks, fuzzy logic methodologies, evolutionary algorithms). His research work has resulted in more than 100 publications in QSAR, modelling algorithms, process control, artificial intelligence and related fields.

Georgia Melagraki (GM) received her Diploma and Ph.D. degrees in Chemical Engineering from NTUA. She has also received the M.Sc. degree in Computational Mechanics and pursued management studies towards an MBA in the same institution. She has a strong scientific background in the field of cheminformatics, QSAR and related fields. Her scientific work has been published in more than 20 original research articles in international peer-reviewed journals.

Andreas Afantitis (AA) received his Diploma and Ph.D. degrees in Chemical Engineering from NTUA. He has also received the M.Sc. degree in Computational Mechanics and pursued management studies towards an MBA in the same institution. Currently he is the director of NovaMechanics Ltd, being responsible for the overall management, strategic direction, growth and financial control. His main research directions are in cheminformatics, bioinformatics and medicinal chemistry. He is a co-author in more than 20 papers in international peer-reviewed journals,

Pantelis Sopasakis (PS) received his Diploma in Chemical Engineering from NTUA and currently he is a Ph.D. student. His research interests are in dynamic modelling, optimal control and stochastic optimization with emphasis on physiological and biological systems.

David Gallagher (DG) has 18 years of human graphical user interface design (GUI) as part of product marketing for computational chemistry SW programs and QSAR tools, with emphasis on the non-expert user. Products include "CAChe WorkSystem" and "ProjectLeader", currently marketed by Fujitsu Ltd. He has published peer-reviewed research papers on QSAR, given oral research presentations on QSAR at ACS and other scientific meetings, led numerous training workshops on QSAR, and created and published tutorials for QSAR training.

Vladimir Poroikov (VP), Prof. Dr., Head of Department for Bioinformatics and Laboratory for Structure-Function Based Drug Design. Member of Editorial Board of several International scientific journals, Chairman of Russian Section of The QSAR and Modelling Society, Member of American Chemical Society and International Society on Computational Biology. Co-author of more than 120 published works and 12 non-open published reports in R&D of new pharmaceuticals, member of the organizing committees and/or invited speaker of many international conferences. VP is a co-investigator of several international projects supported by FP6, FP7, ISTC, INTAS, IFTI, and RFBR.

The Principal Investigator of the MaxTox project, Dr. Indira Ghosh (IG) - Dean and Professor in School of Information Technology, JNU (New Delhi), and Scientific Advisor of SL - has more than a decade of experience working in the pharmaceutical industry (AstraZeneca R&D, Bangalore, India). Before joining AstraZeneca, she obtained her Ph.D. from the prestigious Indian Institute of Science, Bangalore in the field of molecular biophysics. After completing her Ph.D., she accepted a post-doctoral appointment at the University of Houston, Texas with Prof. J. A. McCammon (currently at University of California San Diego, USA).

Sunil Chawla (SC), is the founding director of SL and developed the market for computational chemistry tools in India and California He served as a Market Development Manager for Apple in the USA and was responsible for development of the market for Apple Macs in Scientific/Engineering Markets in the USA, and new products for collaborative learning and new media devices He obtained an M.S. in Biomedical Engineering from McGill University, Montreal, an MBA from UC Berkeley and a B.Tech (EE) from IIT Kharagpur.

Sylvia Escher (SE) is group leader in QSAR and databases in the department of Chemical Risk Assessment at the Fraunhofer Institute of Toxicology and Experimental Medicine (FhG ITEM). The focus of her current work is the development of the RepDose database. Within the OpenTox project she is developing ontologies and Use Cases for repeated dose toxicity.

## 11. Appendices

### Appendix 1: Standards of relevance for OpenTox

Minimum Information Standards for Biological Experiments

http://en.wikipedia.org/wiki/Minimum_Information_Standards

Example standards and formats:

• Minimum Information for Biological and Biomedical Investigations (MIBBI) http://mibbi.sourceforge.net/

• Functional Genomics Experiment (FuGE) http://fuge.sourceforge.net/

• MAGE http://www.mged.org/index.html

• MIAPE http://www.psidev.info/index.php?q=node/91

• Predictive Model Markup Language (PMML) http://www.dmg.org/pmml-v3-0.html

### Toxicity Data

• DSSTox http://www.epa.gov/ncct/dsstox/

• ToxML http://www.leadscope.com/toxml.php

• PubChem http://pubchem.ncbi.nlm.nih.gov/

• OECD Harmonised Templates http://www.oecd.org/document/13/0,3343,en_2649_34365_36206733_1_1_1_1,00.html

• IUCLID5 templates

### Validation

#### Algorithm Validation

• Common best practices such as k-fold cross validation, leave-one-out, scrambling

#### (Q)SAR Validation (Model Validation)

• OECD Principles http://www.oecd.org/dataoecd/33/37/37849783.pdf

• QSAR Model Reporting Format (QMRF) http://qsardb.jrc.it/qmrf/help.html

• QSAR Prediction Reporting Format (QPRF) http://ecb.jrc.it/qsar/qsar-tools/qrf/QPRF_version_1.1.pdf

#### Reports

• REACH Guidance on Information Requirements and Chemical Safety Assessment http://guidance.echa.europa.eu/public-2/getdoc.php?file=information_requirements_en

◦ Part F - Chemicals Safety Report http://guidance.echa.europa.eu/docs/guidance_document/information_requirements_part_f_en.pdf?vers=20_08_08

◦ Appendix Part F http://guidance.echa.europa.eu/docs/guidance_document/information_requirements_appendix_part_f_en.pdf?vers=20_08_08

### Appendix 2: Required Functionality for OpenTox Components

Prediction

**create model ***not applicable in all cases (e.g. expert systems), but required for validation*

*Input *training structures, training activities

*Output *prediction model

predict

*Input *chemical structure, prediction model

*Output *prediction, confidence, supporting information

Descriptor Calculation

calculate

*Input *chemical structure, property

*Output *descriptor(s)

Data Access

create

*Input *new data

update

*Input *modified data

query

*Input *chemical structure, endpoint

*Output *experimental measurement(s)

delete

*Input *ID

Validation

validate

*Input *prediction model, validation method

*Output *validation statistics, supporting information

Report Generation

create report

*Input *data, report type

*Output *report

## Supplementary Material

Additional file 1 **Definition of Ontology**. Description of ontology and vocabulary definitions. Click here for file

Additional file 2 **User Requirements by User Type**. User requirements for several different kinds of OpenTox user are described. Click here for file

Additional file 3 **Interfaces and REST services**. Description of approach to OpenTox interfaces and REpresentational State Transfer (REST) web service architecture. Click here for file

Additional file 4 **Data Schema**. Descriptions of OECD-HT and ToxML data schemas of relevance to OpenTox and the mapping of data resources to the schema. Click here for file

Additional file 5 **OpenTox Algorithm Template**. The fields of the OpenTox description table for the algorithm template are described. Click here for file

Additional file 6 **Initial Implemented OpenTox Algorithms**. Descriptions of initial implemented OpenTox Algorithms for descriptor calculation, classification and regression, clustering and feature selection. Click here for file

Additional file 7 **Validation Use Case Examples**. Description of three example validation Use Cases for application to predictive toxicology models. Click here for file

Additional file 8 **Validation Interfaces and Services**. Description of API for OpenTox Validation services. Click here for file

Additional file 9 **Model Validation Use Case**. Description of Model Validation Use Case execution by OpenTox Web Services. Click here for file

Additional file 10 **Information included in OpenTox Reports**. Description of Information included in OpenTox Reports. Click here for file

Additional file 11 **OpenTox Reporting API and Supported Templates**. Description of reporting formats supported by OpenTox. Click here for file

Additional file 12 **OpenTox Data Infrastructure**. Description of data resources included in initial OpenTox Data Infrastructure. Click here for file

Additional file 13 **Graphical Interface Description of ToxPredict Application Steps**. Description of graphical user interface interactions for steps involved in execution of ToxPredict Application. Click here for file

Additional file 14 **Graphical Interface Description of ToxCreate Application Steps**. Description of graphical user interface interactions for steps involved in execution of ToxCreate Application. Click here for file
