# Supplementary material for: Collaborative development of predictive toxicology applications
Source: J Cheminform. 2010 Aug 31;2:7. doi: 10.1186/1758-2946-2-7 (PMC2941473; doi:10.1186/1758-2946-2-7)
Supplement: Additional file 1 — Definition of Ontology. Description of ontology and vocabulary definitions. [file 1758-2946-2-7-S1.DOC]

**5.1 Additional File 1: Definition of Ontology**

Ontologies are a form of knowledge representation about the world or some part of it [72]. In computer and information science, an ontology is a formal representation of a set of concepts within a domain, and the relationships between those concepts. Within this practical perspective the construction of an ontology consists of: a) selection of entities or objects to be included; b) definition of a controlled vocabulary; c) recognition of relationships and hierarchies, when existing.

Ontologies address questions concerning what entities exist or can be said to exist, and how such entities can be grouped, related within a hierarchy, and subdivided according to similarities and differences [72]. An ontology is used to define and reason about the properties of a domain; thus, an ontology is a "formal, explicit specification of a shared conceptualisation". An ontology provides a shared, or controlled vocabulary, which can be used to model a domain – i.e., the type of objects and/or concepts that exist, and their properties and relations. The Ontology-based informatics approaches provide very powerful tools to organize and retrieve information [73], [74]. The controlled vocabulary is a collection of preferred terms that are used to assist in more precise retrieval of content. Controlled vocabulary terms can be used for categorizing content, building labelling systems, and creating style guides and database schemas. One type of a controlled vocabulary is a taxonomy.

**References**

[72] **Wikipedia Article on “Ontology”** [<http://en.wikipedia.org/wiki/Ontology> ]

[73] McGuinness DL: **Ontologies come of age.** In *Spinning the semantic web: bringing the World Wide Web to its full potential*. Edited by Fensel D, Hendler J, Lieberman H, and Wahlster W: MIT Press, Boston; 2003

[74] **Wikipedia Article on the term “Ontology” in the field of Information Science** [<http://en.wikipedia.org/wiki/Ontology_(information_science)>]
