# Supplementary material for: Collaborative development of predictive toxicology applications
Source: J Cheminform. 2010 Aug 31;2:7. doi: 10.1186/1758-2946-2-7 (PMC2941473; doi:10.1186/1758-2946-2-7)
Supplement: Additional file 2 — User Requirements by User Type. User requirements for several different kinds of OpenTox user are described. [file 1758-2946-2-7-S2.DOC]

**5.2 Additional File 2: User Requirements by User Type**

User requirements for several different kinds of OpenTox user are described here.

**5.2.1 Algorithm Developer (Computer Scientist / Cheminformatician) User**

**User Objective**

The user wishes to evaluate the performance of a newly developed algorithm, with regard to different endpoints.

**Description**

To evaluate the performance of a novel algorithm, it is essential that this algorithm can be compared in predictive performance with regard to other algorithms as well as with regard to different endpoints. For this purpose, a number of potential interesting endpoints have to be selected and a number of standardized k-fold cross validation runs have to be performed. Here, it is important, that the same folds are employed in order to be able to compare algorithm performance using statistical tests. Furthermore, the framework should provide detailed results about applicability domains of the algorithms as well as general overviews on predictive performance. Finally the algorithms should be compared with an independent external test set.
**Requirements**

- provide datasets for a number of endpoints
- provide access to standardized folds
- provide detailed description of predictive performance
- provide summary of comparison to other algorithms

- support testing and comparison against external test set

**5.2.2 Scientific Researcher (Computational Chemist / Toxicologist) User**

**Objective**

The user goal is to construct (Q)SAR Models for toxicity prediction with consideration given to toxicological mechanisms.

**Description**

The user wants to construct and potentially evaluate (Q)SAR models for a small set of specific compounds. This user is concerned with an in-depth analysis for a specific endpoint, covering only a small area of chemical space. The interest lies in the understanding of the underlying concept, and is more concerned with the quality of the model, not the time needed to construct it.

**Requirements**

- provide easy storage access for datasets
- provide the ability to generate new descriptors and features
- provide access to well-tested classification and regression algorithms for prediction
- provide detailed description of reasoning (model)
- provide ability to compare different models

**5.2.3 Early Drug Candidate Screener User**

**Objective**

The user objective is to obtain predictive toxicology model predictions as a component of a drug discovery screening strategy.

**Description**

The user wants to deploy fast algorithms for selection of potential drug candidates guided by most promising directions in chemistry, e.g., retaining activity while avoiding toxicology liabilities.

**Requirements**

- Provide fast active/inactive predictions for a number of different toxicology endpoints
- Understandability and rigour of model and mechanism is not the primary issue cf. performance, confidences and reliability as components of a best practice
- Scalable deployment into virtual screening workflows for libraries of compounds of different sizes

**5.2.4 Risk Assessor User**

**Objective**

The user wants to analyze the risk of compounds being toxic, carcinogenic or mutagenic.

**Description**

The risk assessor wants to make use of a reliable and user-friendly system to evaluate the safety risk of compounds according to endpoints defined by industry and regulatory standards such as REACH, in a time-effective and dependable manner.

**Requirements**

- Provide reliable active/inactive predictions for a number of different endpoints
- Provide model transparency, validation results and statistics
- Provide reports according to required formats
