# Supplementary material for: Collaborative development of predictive toxicology applications
Source: J Cheminform. 2010 Aug 31;2:7. doi: 10.1186/1758-2946-2-7 (PMC2941473; doi:10.1186/1758-2946-2-7)
Supplement: Additional file 3 — Interfaces and REST services. Description of approach to OpenTox interfaces and REpresentational State Transfer (REST) web service architecture. [file 1758-2946-2-7-S3.DOC]

**5.3 Additional File 3: Interfaces and REST services**

An interface is a boundary across which two independent entities meet and interact or communicate with each other. Documenting an interface consists of naming and identifying it and documenting its syntactic and semantic information. The first two parts constitute an interface's "signature". When an interface's resources are invokable by programs, the signature names the programs and defines their parameters. Parameters are defined by their order, data type, and (sometimes) whether or not their value is changed by the program. A signature is the information that you would find about the program, for instance, in an element's C or C++ header file or in a Java interface. An interface is documented with an interface specification, which is a statement of element properties the architect chooses to make known. The architect should expose only what is needed to interact with the interface.

All current OpenTox interfaces are implemented as web services that adhere to the REpresentational State Transfer **(**REST**)** web service architecture [24] for sharing data and functionality among loosely-coupled, distributed heterogeneous systems. The REST architecture is based on five key principles:

1. Every resource can be uniquely identified;
2. Use standard HTTP;
3. Allow multiple representations of resources;
4. Use hypertext links for linking of resources;
5. Communicate statelessly.

Adhering to these principles, the REST web service architecture has a number of desired advantages when compared to other web service architectures:

1. It is lightweight, as only some additional xml mark-up is required;
2. The produced results are human readable, i.e. the resources are uniquely identified by URIs and described by representations;
3. RESTful web services are typically stateless and scalable;
4. The produced web services have a uniform interface (the only allowed operations are the HTTP operations);
5. Components manipulate resources by exchanging representations of the resources.
