# Supplementary material for: Collaborative development of predictive toxicology applications
Source: J Cheminform. 2010 Aug 31;2:7. doi: 10.1186/1758-2946-2-7 (PMC2941473; doi:10.1186/1758-2946-2-7)
Supplement: Additional file 4 — Data Schema. Descriptions of OECD-HT and ToxML data schemas of relevance to OpenTox and the mapping of data resources to the schema. [file 1758-2946-2-7-S4.DOC]

**5.4 Additional File 4: Data Schema**

We describe here OECD-HT and ToxML data schemas of relevance to OpenTox and the mapping of data resources to the schema.

**5.4.1 The OECD Harmonized Templates**

The OECD-HTs correspond to the IUCLID5 XML schemas, which are meant to be used by industry when submitting safety assessment documentation on their chemicals to EU authorities. For each endpoint, the OECD-HTs define a series of fields: e.g., for Template 72, Carcinogenicity, this includes high level fields Administrative data, Materials and Methods, Results and Discussion, Overall Remarks and Attachments, and their associated sub-fields. Since they are generic enough to be able to include data on endpoints with different characteristics, in principle the OECD-HTs provide a substantial basis for building an ontology. However, they are not very formalized and they leave much space to free text entering.

**5.4.2 ToxML (Toxicology XML standard) schema**

ToxML is a public initiative initiated by scientists at Leadscope, Inc [7] to promote adoption and use of controlled vocabularies and XML schema for storing chemical toxicity data. ToxML has the two-fold objective of: a) supporting broadly encompassing and meaningful representations of toxicology experiments, with hierarchical schemes including various levels of complexity; and b) indexing the data with the chemical structures, so as to permit the widest range of chemical biological interrogations of the database [75], [76].

**5.4.3 Data Mapping to Schemas**

A number of analyses were performed mapping a number of databases to the ToxML and OECD-HT schema. The ISSCAN carcinogenicity database was fully mapped to ToxML’s XSD schema and partially to the OECD-HTs schema. Additional mapping exercises included those for aquatic toxicity (EPAFHM [77] in DSSTox), repeated doses toxicity (FhG ITEM’s RepDose), endocrine disruptors (NCTRER [78] in DSSTox), and a second carcinogenicity database (CPDBAS [79] in DSSTox). The ISS *In vivo* micronucleus and Bacterial mutagenesis databases and the FhG ITEM RepDose database were fully mapped to ToxML XSD schema, with in each case valid XML documents (against ToxML XSD schema) obtained.

In principle, both schemas can be used, and both have pros and cons. ToxML seems to be closer to the needs of building databases aimed at scientific computing, but adaptations and extensions for future development may be necessary. OECD-HTs seem to be more suitable for textual archives than for scientific computing. Its main advantage is that it contains schemas for all the various endpoints of regulatory relevance, and there is a rich documentation (Schematron, etc.). In addition, OECD-HTs are already adopted by the important IUCLID5 regulatory database at EChA. Adaptations and extensions seem to be necessary also here. However our analyses showed that due to the volume and level of complexity of OECD-HTs that much more time and effort would be required to adapt it to the modelling needs of the OpenTox data infrastructure. We hence decided to adopt ToxML as the schema for data management and integration within OpenTox, and to support conversion and export to the OECD-HTs for reporting purposes.

The latest version of the ToxML public schema includes many more toxicity studies, than the previous version available before April 1st, 2009. For example, the newest version of the public ToxML schema includes carcinogenicity study type. In addition the latest version has a more extensive documentation for each study type, and the possibility of using both complete schema and separate schemas for each study type.

This analysis showed that there are in principle no constraints to use the ToxML schema to store data from the RepDose, ISSCAN, Ames mutagenicity and *in vivo* micronucleus databases. However, the ontology discussion is not solved only by the mapping of fields of different databases. For example, for repeated dose toxicity it is crucial to also agree on a standardised glossary on target organs and effects.

**References**

[7] **Leadscope’s ToXML schema** [<http://www.leadscope.com/toxml.php>]

[75] Richard AM, Yang C, Judson RS: **Toxicity Data Informatics: Supporting a New Paradigm for Toxicity Prediction.** *Toxicol Mech Method* 2008, **18**:103-11.

[76] **Distributed Structure-Searchable Toxicity (DSSTox) Public Database Network: Coordinating Public Efforts** [<http://www.epa.gov/ncct/dsstox/CoordinatingPublicEfforts.html>]

[77] **Distributed Structure-Searchable Toxicity (DSSTox) Public Database Network: EPAFHM: EPA Feathed Minnow Acute Toxicity Database** [<http://www.epa.gov/ncct/dsstox/sdf_epafhm.html>]

[78] **Distributed Structure-Searchable Toxicity (DSSTox) Public Database Network: NCTRER: FDA National Center for Toxicological Research Estrogen Receptor Binding Database** [<http://www.epa.gov/ncct/dsstox/sdf_nctrer.html>]

[79] **Distributed Structure-Searchable Toxicity (DSSTox) Public Database Network: CPDBAS: Carcinogenic Potency Database Summary Tables – All Species** [<http://www.epa.gov/NCCT/dsstox/sdf_cpdbas.html>]
