# Supplementary material for: Collaborative development of predictive toxicology applications
Source: J Cheminform. 2010 Aug 31;2:7. doi: 10.1186/1758-2946-2-7 (PMC2941473; doi:10.1186/1758-2946-2-7)
Supplement: Additional file 5 — OpenTox Algorithm Template. The fields of the OpenTox description table for the algorithm template are described. [file 1758-2946-2-7-S5.DOC]

**5.5 Additional File 5: OpenTox Algorithm Template**

The fields of the OpenTox description table for the algorithm template are described here.

**Input, Output, Input format and Output format**

Those four fields are used to describe the semantic input and output of the algorithm as well as the file formats for input and output that can be used with the suggested or described implementation of the algorithm.

**User-specified Parameters and Reporting information**

The user-specified parameters are the parameters that have to be or can be adjusted to configure the algorithm. Standard parameters like input or output file name should not be stated here. The reporting information is the algorithm (implementation) output including available statistics and reports.

**Background**

Here the publication date, the popularity in the (Q)SAR and toxicology community, the level of familiarity of (Q)SAR users with the algorithm, the rationale of the approach and further comments on the background of the method/algorithm can be noted.

**Type of Descriptor**

This field is exclusive for descriptor calculation algorithms. It should be filled with a description or explanation of the type of descriptor(s) that are calculated, e.g. physico-chemical or substructural descriptors. Furthermore, comments on the expressiveness and the suitability for similarity and/or distance calculations can be made.

**Applicability Domain/Confidence in Prediction**

This field is exclusive for the classification and regression algorithms. The OECD *guidance document on the validation of (quantitative) structure-activity relationships models* [10] states in paragraph 93 of chapter 3 (“Guidance on principle of a defined domain of applicability”) that a (Q)SAR should be associated with a defined domain of applicability. As the grasp of the concept of applicability domain (AD) is not completely formally defined, we will briefly introduce how AD is used here. Informally, AD is restricted to what is seen on the input and output side during training. A further definition of AD which is also used by the OECD is the following:

*“The applicability domain of a (Q)SAR model is the response and chemical structure space in which the model makes predictions with a given reliability.”*

Furthermore, OECD advises that the AD principle should be applied in a model-specific manner. Thus, every model should be associated with its own AD derived not only on the chemicals in the training set but also on the descriptors and (statistical) approach used to develop the model. Ideally, the AD should be defined and documented by the model developer. Consequently it only makes sense to apply the concept of AD to the second domain of algorithms, namely the classification and regression algorithms, which will be used in OpenTox to derive the (Q)SAR models. Apart from the composition of the training set and the initially calculated descriptors, the methods' inherent bias and methodology has an influence on the AD of the resulting model, as they have an effect on the model’s response space.

Related to the concept of an AD is the concept of a confidence in predictions inherent in most machine learning algorithm. Clearly, most modern machine learning algorithms do not only provide a categorical class label, but also a probability with which the class is predicted. The confidence in predictions comes in many flavors e.g., margins but in most cases it can be transformed back into probability estimates (in the case of margins by methods like Platt scaling). Most considerations concerning *abstaining* from prediction in the machine learning literature are centered around the confidence in predictions. The main difference is that the confidence is only known when the model is already applied, i.e., in hindsight, whereas the applicability domain seems to be defined for the input space directly. As both concepts are obviously related, statements about the applicability as well as about the confidence in predictions can be entered in this field of the template.

**Bias, lazy/eager Learning and Interpretability of Models**

These three fields are exclusive for the classification and regression algorithms. They contain information if the algorithm has an intrinsic bias, e.g. feature-selection bias or instance-selection bias. Furthermore it is stated if the method is an eager or a lazy learning method. The third field contains information of how easy it is to interpret the model or if the algorithm learns or involves complete black box models.

**Class-blind/Class-sensitive Feature Selection**

This field is exclusive for feature selection algorithms. It contains information if the algorithm selects the features class-blind or class-sensitive.

**Type of Feature Selection and of Approach**

These are two fields exclusive for feature selection algorithms. The type of feature selection algorithm is either an optimal, a greedy or a randomized algorithm. The type of the approach is either a filter, a wrapper or a hybrid approach.

**Performance**

This field gives information on the algorithm’s performance regarding time and space usage. Exemplary running times and memory consumption can be stated as well as theoretical considerations.

**OpenTox Availability, License/Dependencies**

In these two fields the availability of the algorithm/implementation to the OpenTox Framework is explained. In the license and dependencies field information about the license the implementation is published under and about other software packages the implementation is dependent on are gathered.

**Convenience of Integration and Priority**

The convenience of integration field gives information about how easy it will be to integrate the software into the OpenTox Framework. Relevant are for example, if the implementation is dependent on a specific operating system or not, or if parts of it have to be adjusted before integration. The priority (divided into three categories *A*, *B* and *C*) is not to be understood as prescriptive but just as a guidance for overall development planning.

**Author of Method, Author of Description, Contacts and Comments**

The last fields are used to facilitate the communication regarding the algorithms. The first field shall be filled with the name(s) of the author(s) of the algorithm/implementation and the contact email address. The second field states the author who filled the description table and the contact within OpenTox gives a contact email address within the OpenTox community. The remaining comments field can be used for any further comment on the method including reviews.

**Algorithm Documentation and Submission**

Accepted algorithms documented accorded to the above template are published through the developer area of the website [22]. Algorithm developers in the community may submit further algorithms for potential inclusion in the Framework and development planning using the template format.

**References**

[10] **OECD Validation Principles** [<http://www.oecd.org/dataoecd/33/37/37849783.pdf>]

[22] **OpenTox Development: The Developers’ Area of the OpenTox Project** [<http://www.opentox.org/dev>]
