# Supplementary material for: Collaborative development of predictive toxicology applications
Source: J Cheminform. 2010 Aug 31;2:7. doi: 10.1186/1758-2946-2-7 (PMC2941473; doi:10.1186/1758-2946-2-7)
Supplement: Additional file 6 — Initial Implemented OpenTox Algorithms. Descriptions of initial implemented OpenTox Algorithms for descriptor calculation, classification and regression, clustering and feature selection. [file 1758-2946-2-7-S6.DOC]

**5.6 Additional File 6: Initial Implemented OpenTox Algorithms**

Algorithm web services are key components of the overall OpenTox Framework and also important parts of Use Case-related implementations, as they are responsible for data manipulation, descriptor calculation and selection, reduction of dimensionality, and most importantly generation of regression and classification (Q)SAR models. All algorithm services accept a dataset as input, so they assume the existence of a dataset service. Algorithms that have been included in the initial prototype are summarised here and can be grouped in four categories: descriptor calculation algorithms, classification and regression algorithms, clustering algorithms and feature selection algorithms.

**5.6.1Descriptor Calculation Algorithms**

This category currently includes algorithms which calculate descriptors that represent chemical structures. There are two different types of molecular descriptors, namely physico-chemical and (sub-)structural descriptors. In the group of structural and sub-structural descriptors, five algorithms have been implemented (FreeTreeMiner, fminer, gSpan [80,81], MakeMNA [82], MakeQNA [83]). We have also prototyped two sets of descriptor calculation algorithms that belong to the group of physico-chemical descriptors, namely the Chemistry Development Toolkit (CDK) and JOELib [84], [85]. OpenBabel has not been implemented yet as a separate software component, but it is implicitly used in the fminer descriptor calculation implementation for sub-structure matching.

**5.6.2 Classification and Regression Algorithms**

These services are responsible for the generation of QSAR models which are stored on the server side and can be used for predicting toxicological properties. They require the specification of a dataset URI and of a URI for the prediction feature. Some algorithms accept additional parameters that allow the fine tuning of the training procedure.

For the initial OpenTox prototype we have implemented MLR as a basic regression method [86], kNN [87] as a basic instance-based lazy learning classification method, J48 decision trees as an eager classification algorithm implementation [88], PLS [89], SVM [90], lazar, ToxTree [91], Gaussian processes for regression [92], M5P [93], and MaxTox [94]. Additionally, we have implemented one popular machine learning classification algorithm, namely the Naive Bayes classification method.

**5.6.3 Clustering Algorithms**

This category contains unsupervised learning algorithms that group objects of similar kind into respective categories. In other words clustering algorithms are exploratory data analysis tools which aim at sorting different objects into groups in a way that the degree of association between two objects is maximal if they belong to the same group and minimal otherwise. The most popular clustering algorithm, namely the k-means clustering method [95] has been implemented in the initial prototype.

**5.6.4 Feature Selection Algorithms**

The fourth category contains algorithms for the reduction of the dimensionality of a dataset, by selecting only a subset of a full set of descriptors included in the dataset. The feature selection algorithm Info Gain Attribute Evaluation [96] has been included in the initial prototype.

It should be noted that all algorithm services are required to adhere to the OpenTox Algorithm API [97], regardless of the category to which they belong. Further information on each algorithm that has been prototyped as a web service can be located on the OpenTox website [98].

**References**

[80] Yan X, Han J: **gSpan: Graph-based substructure pattern mining.** In *Proceedings of the 2002 IEEE international Conference on Data Mining: December 09-12, 2002; Maebashi, Japan*. ICDM. IEEE Cmputer Society, Washington, DC; 2002:721-724.

[81] Jahn K, Kramer S: **Optimizing gSpan for Molecular Datasets**. In *Proceedings of the Third International Workshop on Mining Graphs, Trees and Sequences (MGTS-2005).* Edited by Nijssen S, Meinl T, Karypis, G.; 2005:77-89

[82] **MakeMNA Descriptor Calculation** [<http://www.opentox.org/dev/documentation/components/makemna>]

[83] **MakeQNA Descriptor Calculation** [<http://www.opentox.org/dev/documentation/components/makeqna>]

[84] **A Java Based Cheminformatics (Computational Chemistry) Library** [<http://www.ra.cs.uni-tuebingen.de/software/joelib/index.html>]

[85] **Tutorial for the JOELib Java Based Cheminformatics (Computational Chemistry) Library** [<http://www.ra.cs.unituebingen.de/software/joelib/tutorial/JOELibTutorial.pdf>]

[86] Breiman L, Friedman JH, Olshen RA, Stone CJ: *Classification and Regression Trees*. Belmont, CA: Wadsworth; 1984.

[87] **Wikipedia Article on the k-Nearest Neighbor Algorithm (kNN)** [<http://en.wikipedia.org/wiki/K-nearest_neighbor_algorithm>]

[88] **OpenTox Component: J48 Algorithm** [<http://www.opentox.org/dev/documentation/components/j48>]

[89] **OpenTox Component: Partial-Least Squares Regression (PLS)** [<http://www.opentox.org/dev/documentation/components/pls>]

[90] **Wikipedia Article on Support Vector Machines (SVM)** [<http://en.wikipedia.org/wiki/Support_vector_machine>]

[91] Patlewicz G, Jeliazkova N, Safford RJ, Worth AP, Aleksiev B. **An evaluation of the implementation of the Cramer classification scheme in the Toxtree software**. *SAR QSAR Environ Res* 2008, **19**(5-6):495-524.

[92] Rasmussen CE, Williams CKI: *Gaussian Processes for Machine Learning (Adaptive Computation and Machine Learning)*. The MIT Press; 2005.

[93] **OpenTox Component: M5P** [<http://www.opentox.org/dev/documentation/components/m5p>]

[94] Prakash O, Ghosh I: **Developing an Antituberculosis Compounds Database and Data Mining in the Search of a Motif Responsible for the Activity of a Diverse Class of Antituberculosis Agents**. *J Chem Inf Model* 2006, **46**:17-23.

[95] **Wikipedia Article on K-Means clustering** [<http://en.wikipedia.org/wiki/K-means_clustering>]

[96] Liu H, Setiono R: **Chi2: Feature selection and discretization of numeric attributes**. In *Proceedings of the 7th International Conference on Tools with Artificial Intelligence: November 05-08 1995;* IEEE; 1995:338-391.

[97] **OpenTox Algorithms Ontology** [<http://www.opentox.org/dev/apis/api-1.1/Algorithms>]

[98] **OpenTox Testing Procedures** [<http://www.opentox.org/dev/testing>]
