# Supplementary material for: Collaborative development of predictive toxicology applications
Source: J Cheminform. 2010 Aug 31;2:7. doi: 10.1186/1758-2946-2-7 (PMC2941473; doi:10.1186/1758-2946-2-7)
Supplement: Additional file 7 — Validation Use Case Examples. Description of three example validation Use Cases for application to predictive toxicology models. [file 1758-2946-2-7-S7.DOC]

**5.7 Additional File 7: Validation Use Case Examples**

We describe here three example validation Use Cases for application to predictive toxicology models.

**5.7.1 Validate a Prediction Model**

The user who wants to evaluate a prediction model carries out the following steps:[¶](http://opentox.org/wiki/wp1/Validate_a_prediction_model#Validate-a-prediction-model)

1. User input:[¶](http://opentox.org/wiki/wp1/Validate_a_prediction_model#3A41-User-input)

- Prediction model
- Training structures and activities
- Testing structures and activities or validation algorithm

2. Create test sets with validation algorithm (if no test structures are provided)[¶](http://opentox.org/wiki/wp1/Validate_a_prediction_model#3A42-Create-test-sets-with-validation-algorithm-if-no-test-structures-are-provided)

3. Remove overlapping compounds between training and test sets[¶](http://opentox.org/wiki/wp1/Validate_a_prediction_model#3A43-Remove-overlapping-compounds-between-training-and-test-sets)

4. Create prediction model with training set[¶](http://opentox.org/wiki/wp1/Validate_a_prediction_model#3A44-Create-prediction-model-with-training-set)

5. Predict test set with prediction model[¶](http://opentox.org/wiki/wp1/Validate_a_prediction_model#3A45-Predict-test-set-with-prediction-model)

6. Repeat n-times for n-fold Cross Validation[¶](http://opentox.org/wiki/wp1/Validate_a_prediction_model#3A46-Repeat-n-times-for-n-fold-CV)

7. Display summary statistics[¶](http://opentox.org/wiki/wp1/Validate_a_prediction_model#3A47-Display-summary-statistics)

**5.7.2 Integrating and Validating a new Algorithm / Model**

The user carries out the following steps:**[¶](http://opentox.org/wiki/wp1/Integrating_and_validating_a_new_Algorithm#3A5-Integrating-and-validating-a-new-algorithm-model)**

1. Implement algorithm according to interface specification[¶](http://opentox.org/wiki/wp1/Integrating_and_validating_a_new_Algorithm#3A51-Implement-algorithm-according-to-interface-specification)

Developer needs

- interface specification
- possibility to specify which parameters are needed by his algorithm

2. Integrate algorithm into the framework[¶](http://opentox.org/wiki/wp1/Integrating_and_validating_a_new_Algorithm#3A52-Integrate-algorithm-into-the-framework)

- Test algorithm by controlling / validation components for correct input and output

3. Validate model[¶](http://opentox.org/wiki/wp1/Integrating_and_validating_a_new_Algorithm#3A53A-Validate-model)

- The above case for Validate a Prediction Model is run

4. Validate model to previous version of the algorithm[¶](http://opentox.org/wiki/wp1/Integrating_and_validating_a_new_Algorithm#3A53B-Validate-model-to-previous-version-of-the-algorithm)

- Allows user to compare a modified version of the algorithms to older versions

5. Compare model to a range of other models / algorithms[¶](http://opentox.org/wiki/wp1/Integrating_and_validating_a_new_Algorithm#3A53C-Compare-model-to-a-range-of-other-models-algorithms)

- The Use Case below for Compare Performance of Different Models/Algorithms is run

**5.7.3 Compare Performance of Different Models / Algorithms**[¶](http://opentox.org/wiki/wp1/Compare_performance_of_different_models__algorithms#3A6-Compare-performance-of-different-models-algorithms)

1. User input:[¶](http://opentox.org/wiki/wp1/Compare_performance_of_different_models__algorithms#3A61-User-input)

- Select models that should be compared
- Select datasets / endpoints
- Select validation routine (cross validation / training-test split)

2. Validate models[¶](http://opentox.org/wiki/wp1/Compare_performance_of_different_models__algorithms#3A62-Validate-models)

- Run Use Case for Validate a Prediction Model
- The validation results may be stored for succeeding runs

3. Display summary statistics[¶](http://opentox.org/wiki/wp1/Compare_performance_of_different_models__algorithms#3A63-Display-summary-statistics)

- The user should be able to select different views on the results
