# Supplementary material for: Collaborative development of predictive toxicology applications
Source: J Cheminform. 2010 Aug 31;2:7. doi: 10.1186/1758-2946-2-7 (PMC2941473; doi:10.1186/1758-2946-2-7)
Supplement: Additional file 8 — Validation Interfaces and Services. Description of API for OpenTox Validation services. [file 1758-2946-2-7-S8.DOC]

**5.8 Additional File 8: Validation Interfaces and Services**

A Validation API is included in the OpenTox APIs ensuring the seamless interaction between all OpenTox components with regards to validation needs. Each validation resource for example, contains information about the dataset and the model, so the underlying procedures can be invoked.

We use the notation ‘/resource’ to denote the class of URIs someDomain.com/resource where someDomain.com can be the domain name of any OpenTox server (such as opentox.informatik.uni-freiburg.de). We use sub-URIs to distinguish different web services: e.g. opentox.informatik.uni-freiburg.de/validation for the validation web service. All validation resources share this prefix. For example [opentox.informatik.uni-freiburg.de/validation/1](http://opentox.informatik.uni-freiburg.de/validation/1) is the result of a plain test-set validation with ID 1, [opentox.informatik.uni-freiburg.de/validation/2](http://opentox.informatik.uni-freiburg.de/validation/2) is the resource of a cross-validation with ID 2.

The validation API consists of a number of operations that use one of the following REST methods: GET, PUT, POST, or DELETE [99]:

1. GET /: Returns a list of all validations on the server. Currently supported return formats are: text/uri-list
2. GET / {id}: Returns a representation of a specific validation object, identified by its id. The requested MIME type should be set in the requests "Accept" header, e.g. ‘curl -X GET -H "Accept:application/xml" http://{server}/validation/{id}’. The supported MIME types are: application/rdf+xml and text/x-yaml (default).
3. POST /: Validates a model on a test dataset. Two different versions are provided:
   - The first version validates an already existing model. It requires the parameters ‘model_uri’, ‘test_dataset_uri’ and ‘prediction_feature’. The result of such an operation is a task URI, as the validation process will be executed asynchronously. As soon as the validation is finished the task will provide the validation URI.
   - The second version creates a model with a training dataset first, then validates the model on a test dataset. The model building step is automatically performed if the given parameters are ‘algorithm_uri’, ‘prediction_feature’, ‘algorithm_params’, ‘training_dataset_uri’, and ‘test_dataset_uri’. The result is again a task, that links to the validation resource as soon as it is finished. In case the user wants to compare a model to models trained on a y-scrambled dataset ([www.qsarworld.com/qsar-statistics-y-scrambling.php](http://www.qsarworld.com/qsar-statistics-y-scrambling.php)), the optional ‘y_scramble’ (default=false) and ‘y_scramble_seed’ (default=1) can be used.
4. POST /training_test_split is similar to the operation just described, but splits one single dataset into training and test sets and then performs model construction on one part of the dataset while evaluating the learned model on the remainder of the original dataset. The required parameters are as follows: ‘algorithm_uri’, ‘prediction_feature’, ‘algorithm_params’, ‘dataset_uri’, ‘split_ratio’ (default=0.66), ‘random_seed’ (default=1), and the optional ‘y_scramble’ and ‘y_scramble_seed’. The result is again a validation URI or a task URI.
5. DELETE /{id} deletes a specified validation object.

The same design concepts were used in the construction of the Cross-Validation API. A cross-validation component performs k single validations using a standard k-fold cross-validation.

1. GET /crossvalidation: Returns a list of all cross-validations on the server. Currently supported return formats are: text/uri-list
2. GET /crossvalidation/{id}: Returns a representation of a specific cross-validation object, identified by its id. The requested MIME type should be set in the requests "Accept" header. The supported MIME types are: application/rdf+xml and text/x-yaml (default).
3. GET /crossvalidation/{id}/validations: Returns a list of validation objects associated with a specific cross-validation object, identified by its id. The result is a list of validation URIs.
4. POST /crossvalidation: A POST operation on a validation activates the application of a k-fold cross-validation routine, given a given dataset and an algorithm. The result of such an operation is a cross-validation URI or a task URI. The following parameters have to be submitted: ‘algorithnm_uri’, ’prediction_feature’, ’algorithm_params’ (default=""), ’num_folds’ (default=10), ‘random_seed’ (default=1), ‘stratified’ (default=true), ‘y_scramble’ (default=false), and ‘y_scramble_seed’ (default=1).
5. POST /crossvalidation/loo: This performs a leave-one-out cross-validation, resulting in a cross-validation URI or a task URI. In contrast to the normal k-fold cross-validation the parameters for splitting the dataset are not required. The rest of the parameters are the same.
6. DELETE /crossvalidation/{id} deletes a specified validation object.

**Reference**

[99] **Common Methods for HTTP/1.1** [<http://www.w3.org/Protocols/rfc2616/rfc2616-sec9.html>]
