# Supplementary material for: Collaborative development of predictive toxicology applications
Source: J Cheminform. 2010 Aug 31;2:7. doi: 10.1186/1758-2946-2-7 (PMC2941473; doi:10.1186/1758-2946-2-7)
Supplement: Additional file 9 — Model Validation Use Case. Description of Model Validation Use Case execution by OpenTox Web Services. [file 1758-2946-2-7-S9.DOC]

**5.9 Additional File 9: Model Validation Use Case**

Here we provide a description of a Model Validation Use Case execution by OpenTox Web Services. In the example described below, the command line tool curl [100] is used.

The Use Case is executed in 10 steps as follows:

- Step 1: The user invokes the validation with a POST REST operation towards the validation web service, with parameters ‘algorithm_uri’, ‘training_dataset_uri’, ‘test_dataset_uri’, ‘prediction_feature’, and ‘algorithm_params’:

curl -X POST –d algorithm_uri=<http://webservices.in-silico.ch/test/algorithm/lazar> -d training_dataset_uri=<http://opentox.informatik.uni-freiburg.de/dataset/2> -d test_dataset_uri=<http://opentox.informatik.uni-freiburg.de/dataset/3> -d prediction_feature=[http://www.epa.gov/NCCT/dsstox/CentralFieldDef.html#ActivityOutcome_CPDBAS_Hamster](http://www.epa.gov/NCCT/dsstox/CentralFieldDef.html#ActivityOutcome_CPDBAS_Hamster) -d algorithm_params="feature_generation_uri=http://webservices.in-silico.ch/test/algorithm/fminer" <http://opentox.informatik.uni-freiburg.de/validation>

This is the only necessary REST call performed by the user. The subsequent steps are processed internally.

- Step 2: The validation web service starts the model building process, by automatically performing a POST REST operation addressed to the algorithm web service:

curl -X POST -d dataset_uri=<http://opentox.informatik.uni-freiburg.de/dataset/2> -d prediction_feature=[http://www.epa.gov/NCCT/dsstox/CentralFieldDef.html#ActivityOutcome_CPDBAS_Hamster](http://www.epa.gov/NCCT/dsstox/CentralFieldDef.html#ActivityOutcome_CPDBAS_Hamster) -d feature_generation_uri=http://webservices.in-silico.ch/test/algorithm/fminer <http://webservices.in-silico.ch/test/algorithm/lazar>

The algorithm web service fetches the training data and builds a model (Step 3 and 4). This Use Case includes a feature generation process: The fminer service is used to mine structural features that occur in the compounds of the training dataset. The features are stored as a new dataset in the dataset web service, which is required to build the lazar model. Finally, the algorithm web service returns the model URI as result (Actually, a task object is returned first, while the process is running in the background. This is done for all time-consuming processes.).

- Step 5: The validation web service uses the returned model URI to predict the compounds in the validation test set:

curl -X POST –d dataset_uri=http://opentox.informatik.uni-freiburg.de/dataset/3 <http://webservices.in-silico.ch/test/model/<id>>

The model web service therefore fetches the test compounds and makes predictions. The predictions are stored in a new dataset (Steps 6 and 7)

Steps 8 – 10: The validation web service retrieves the predictions from the dataset web service, and computes the validation statistics. In order to save hard-disk space, the prediction dataset can be deleted afterwards. This is an optional setting which is not included yet. Finally, the validation object is available to the user.

**Reference**

[100] **cURL Homepage** [<http://curl.haxx.se/>]
