# Supplementary material for: Collaborative development of predictive toxicology applications
Source: J Cheminform. 2010 Aug 31;2:7. doi: 10.1186/1758-2946-2-7 (PMC2941473; doi:10.1186/1758-2946-2-7)
Supplement: Additional file 10 — Information included in OpenTox Reports. Description of Information included in OpenTox Reports. [file 1758-2946-2-7-S10.doc]

**5.10 Additional File 10: Information included in OpenTox Reports**

Information that should be included in most OpenTox reports is as follows:[¶](http://opentox.org/wiki/wp5/Reporting#General-information-that-should-be-included-in-most-of-the-reports)

- General information
  - Date
  - Runtime
  - User (if available)
  - Job description (what was done, endpoint)
- Description of the data
  - CAS number
  - Smiles string
  - Inchi code
- Description of the features used
  - What kind of features
  - Which/How many features have been used
  - How have the features been generated
- Description of the model(s)
  - Which model was used (how does it work)
  - How the model was trained
    - Data used for training
    - Training performance
- Prediction Results
  - Activity
  - Some confidence measurement, if possible
  - Applicability domain
  - Graphs/Diagrams
  - Decisive features / rules / components
- Model validation results
  - Descriptions of the routines used
  - Confusion matrix
- Warnings (in case of inappropriate usage)
  - Parameter fiddling
  - Too few training examples, etc...
- Additional Information for the inexperienced user
  - Explanation of QSAR prediction
  - Guidance on results interpretation
