# Supplementary material for: Collaborative development of predictive toxicology applications
Source: J Cheminform. 2010 Aug 31;2:7. doi: 10.1186/1758-2946-2-7 (PMC2941473; doi:10.1186/1758-2946-2-7)
Supplement: Additional file 11 — OpenTox Reporting API and Supported Templates. Description of reporting formats supported by OpenTox. [file 1758-2946-2-7-S11.DOC]

**5.11 Additional File 11: OpenTox Reporting API and Supported Templates**[¶](http://opentox.org/wiki/wp5/Reporting#Reports-following-different-standards-templates)

**5.11.1 QSAR Model Reporting Format (QMRF)**[¶](http://opentox.org/wiki/wp5/Reporting#QSAR-Model-Reporting-Format-QMRF)

Developers and users of (Q)SAR models can submit to the JRC information on (Q)SARs by using the (Q)SAR Model Reporting Format [5]. QMRF is a harmonised template for summarizing and reporting key information on (Q)SAR models, including the results of any validation studies. The information is structured according to the OECD (Q)SAR validation principles. A DTD XML schema is used to generate reports.

**5.11.2 QSAR Prediction Reporting Format (QPRF)**[¶](http://opentox.org/wiki/wp5/Reporting#QSAR-Prediction-Reporting-Format-QPRF)

The QSAR Prediction Reporting Format (QPRF) is a harmonised template for summarizing and reporting substance-specific predictions generated by (Q)SAR models [101].

**5.11.3 REACH Chemical Safety Report (CSR)**[¶](http://opentox.org/wiki/wp5/Reporting#REACH-Chemical-Safety-Report-CSR)

The REACH 'registration dossier' is the set of information submitted by a registrant for a particular substance to comply with registration requirements. It consists of two main components:

(i) a technical dossier, which has to be submitted using the IUCLID 5 format

(ii) a chemical safety report, which is a stand-alone document attached in the IUCLID registration dossier.

The main goal of the chemical safety report (CSR) is to document the chemical safety assessment (CSA), including its conclusions and results [102-104].

**5.11.4 OpenTox Reporting API**

The OpenTox Reporting API currently provides the following reporting capabilities for all validated models:

1. GET /report: Retrieves a list of reports types stored on the server. The available report types are described in detail below.
2. GET /report/{report-type}: Retrieves a list of all reports for the particular report type. The result is a list of available reports as URI.
3. GET /report/{report-type}/{id} : Retrieves a specific report for the particular report type in one of the following output formats: XML, PDF, HTML, or RTF. The accept-header is used to differentiate between different output formats.
4. POST /report/{report-type}: Creates a report for the specific report types. The parameters, as well as the results, are report-type specific and are detailed below:
5. POST /report/toxpredict: This creates a ToxPredict report. ToxPredict is one of the initially selected OpenTox prototype Use Cases where the user requires a number of models predicting toxicity for one or a library of compounds. The required parameters are a ‘list of validation URIs’ representing the outcome of the employed models. The result is a report URI or task URI.
6. POST /report/validation: This operation creates a single validation report for one model applied to one dataset. The required parameter is a ‘validation URI’. The result is a report URI or task URI.
7. POST /report/crossvalidation: This creates a cross-validation report for one algorithm applied to one dataset, split into k folds. The required parameter is a ‘crossvalidation URI’. The result is a report URI or task URI.
8. POST /report/algorithm_comparison: This creates a report to compare the performance of different algorithms when applied to the same datasets and cross-validation splits. The report shows which algorithm performs better on the dataset, and compares its overall performance. Again ‘validation URI’s’ or ‘cross-validation’s’ are required as input parameters, with the prerequisite that there is one validation for each algorithm and cross-validation/test dataset provided.
9. POST /report/model_comparison: Creates report for comparing different models, i.e. multiple models applied to one dataset. The required parameter is a ‘list of validation URIs’
10. POST /report/qmrf: Creates a QMRF report [105]. As this kind of reporting format can only partially be filled automatically, it is intended to produce a report in XML or RTF format, which can later be filled either via the QMRF Editor [53] or within a word processing program like OpenOffice Writer [106]. The required parameters are a list of cross-validation URIs and/or validation URIs of the same model. The result is a report URI or task URI.
11. POST /report/qprf: Creates a QPRF report. As with the QMRF reporting format, this can only partially be filled using automated methods. Therefore, we also include relevant fields for manual editing.
12. DELETE /report/{report-type}/{id}: Deletes a specified report based on the report type and id.

**References**

[5] **ECB QSAR Model Reporting Format (QMRF)** [http://ecb.jrc.ec.europa.eu/qsar/qsar-tools/index.php?c=QRF]

[53] **AMBIT QMRF editor** [<http://ambit.sourceforge.net/qmrf/jws/qmrfeditor.jnlp>]

[101] **QSAR Prediction Reporting Format (QPRF)** [<http://ecb.jrc.ec.europa.eu/qsar/qsar-tools/qrf/QPRF_version_1.1.pdf>]

[102] **REACH Guidance on Information Requirements and Chemical Safety Assessment** [<http://guidance.echa.europa.eu/docs/guidance_document/information_requirements_en.htm>]

[103] **ECHA Guidance on Information Requirements and Chemical Safety Assessment, Part F: Chemicals Safety Report** [<http://guidance.echa.europa.eu/docs/guidance_document/information_requirements_part_f_en.pdf?vers=30_07_08>]
[104] **ECHA Guidance on Information Requirements and Chemical Safety Assessment, Appendix Part F, including the template** [<http://guidance.echa.europa.eu/docs/guidance_document/information_requirements_appendix_part_f_en.pdf?vers=30_07_08>]

[105] **QSAR Reporting Formats and JRC QSAR Model Database** [<http://ecb.jrc.ec.europa.eu/qsar/qsar-tools/index.php?c=QRF>]

[106] **OpenOffice - The Free and Open Productivity Suite** [<http://www.openoffice.org/>]
