# Supplementary material for: Collaborative development of predictive toxicology applications
Source: J Cheminform. 2010 Aug 31;2:7. doi: 10.1186/1758-2946-2-7 (PMC2941473; doi:10.1186/1758-2946-2-7)
Supplement: Additional file 12 — OpenTox Data Infrastructure. Description of data resources included in initial OpenTox Data Infrastructure. [file 1758-2946-2-7-S12.DOC]

**5.12 Additional File 12: OpenTox Data Infrastructure**

**5.12.1 Ambit Data Resource**

The initial OpenTox data infrastructure is being supported by Ambit, an open source software for cheminformatics data management. It consists of a database and functional modules allowing for a variety of queries and data mining of the information, stored in the database. Ambit functionality is being extended and wrapped as REST web services within the Framework of the OpenTox project. Detailed information on Ambit (source code, executables and a link to REST services) is available on the Ambit sourceforge website [38]. The Ambit database is a relational database, consisting of several repositories for compounds, properties, QSAR models, users, references, as well as several tables containing pre-processed information which allows speeding up substructure and similarity queries. The current implementation is based on MySQL [107].

**5.12.2 Data Provenance and Quality Assurance**

The Ambit database provides means to identify the origin of the data, i.e., the specific inventory a compound originated from. The correctness of the chemical structure is crucial for QSAR modelling. In order to raise the awareness of possibly incorrect structures that might be imported from external sources, Ambit allows assigning quality labels to each 2D chemical structure, as follows:

- Manual verification by expert(s). Any user can assign quality labels and explain the reason of the assignment (Ambit table *quality_structure*). The reasons can include discrepancies between registry numbers, names and structure, expert knowledge, manual comparison with external sources, etc. The list of quality labels includes:
  - '*OK*' – the structure is correct;
  - '*ProbablyOK*' – most probably the structure is correct, but some issues still need to be verified;
  - '*Unknown*' – not possible to assign a definite label;
  - '*ProbablyERROR*' – most probably there is an error;
  - '*ERROR*' – the structure is definitely wrong.
- Automatically verified, by comparing the structures available under the same chemical compound entry (e.g. imported from different sources):
  - ‘*Consensus*' – all structures under the same chemical compound entry are identical;
  - '*Majority*' – the majority of structures under the same chemical compound entry are the same, but there is a small number of structures, which differ from the majority (e.g. structures come from 3 different sources and two of the three structures are identical);
  - '*Ambiguous*' - there is no majority of equal structures under the same chemical compound entry (e.g. structures come from 3 different sources and all of them are different);
  - '*Unconfirmed’ -* the structure comes from a single source and it is impossible to make a comparison.

Furthermore, the results of these automatic comparisons of structures coming from different sources are used for assigning initial quality labels, subject to further review by experts.

**5.12.3 Selection of Data sources for Inclusion**

Chemical structures and data from various sources have been imported into the Ambit database and made available via the OpenTox REST API interface. The data sources have been selected within publicly available data sources, providing high-quality structural and/or toxicological data:

- ECHA list of pre-registered substances [59] - The original version of this list was created by ECHA on the basis of information submitted by companies before December 1, 2008. Since then, ECHA has done some tidying up - for example, reconciling the numbers and names of chemicals, putting duplications together and making deletions where companies have requested them. According to ECHA, quote “some 14,000 of the entries on the list could contain mistakes as far as we can see - sometimes this might be because the name and number don't match; because of language difficulties; because a mixture of substances was given rather than a single substance; or simply because, for whatever reason, a substance doesn't have a number.” We have decided to include this data source, regardless of its relatively high error rate (estimated at around 10%), because of its paramount importance and relevance in a REACH [102] context;
- Chemical Identifier Resolver [63] - A REST web service evolved from the Chemical Structure Lookup Service [108] and provided by the NCI/CADD group [109] of the US National Cancer Institute. This service works as a resolver for different chemical structure identifiers and allows one to convert a given structure identifier into another representation or structure identifier. The total number of chemical structures known by the service currently is 92,939,226;
- ChemIDplus [64] - a free, web-based search system that provides access to structure and nomenclature authority files used for the identification of chemical substances cited in the US National Library of Medicine (NLM) databases [110], including the TOXNET® system [111]. ChemIDplus also provides structure searching and direct links to many biomedical resources at NLM and on the Internet for chemicals of interest. The database contains over 388,661 chemical records, of which 295,119 include chemical structures and molecular weight, 139,354 have toxicity data, 25,461 have physical property data, and is searchable by Name, Synonym, CAS Registry Number, Molecular Formula, Classification Code, Locator Code, Structure, Toxicity, and/or Physical properties;
- ChemDraw [112] - a molecule editor developed by the cheminformatics company CambridgeSoft [113]. We have used its “Convert Name to Structure” functionality for manual conversion and further checking of some of the chemical compound names included in the above mentioned ECHA list of pre-registered substances;
- JRC PRS list [60] - Contains structures for a subset (80,410) of the above mentioned ECHA list of pre-registered substances, generated by the Computational Toxicology Group, Joint Research Centre [114], through the ACD/Name to structure Batch software [115], which converts systematic and non-systematic chemical names of general organic and select biochemical and inorganic compounds into structures;
- ISSCAN [37] - Istituto Superiore di Sanità (ISS), "CHEMICAL CARCINOGENS: STRUCTURES AND EXPERIMENTAL DATA". This database originates from the experience of researchers of the Environment and Primary Prevention Department in the field of structure-activity relationships (SAR), aimed at developing models which theoretically predict the carcinogenicity of chemicals. A portion of the chemicals has been the subject of carcinogenicity classification by various Regulatory Agencies and Scientific Bodies. The database has been specifically designed as an expert decision support tool and includes these carcinogenicity classification "calls" to guide the application of SAR approaches;
- DSSTox [6] - a project of the US EPA's National Centre for Computational Toxicology [116], helping to build a public data foundation for improved structure-activity and predictive toxicology capabilities. The DSSTox website provides a public forum for publishing downloadable, structure-searchable, standardized chemical structure files associated with toxicity data. In particular, we have included in the OpenTox prototype database the following DSSTox datasets:
  - CPDBAS [79] - Carcinogenic Potency Database Summary Tables - All Species. The CPDB Summary Tables list summarized results for experiments on 1547 substances in the Carcinogenic Potency Database (CPDB). These Summary Tables report the strongest evidence of carcinogenicity for each chemical, in each sex/species and represent one of many possible summarizations of the data in the CPDB. The CPDB includes detailed results and analyses of 6540 chronic, long-term carcinogenesis bioassays reported in 1513 papers in the general literature and 452 Technical Reports of the US National Cancer Institute/National Toxicology Program [36];
  - DBPCAN [117] - EPA Water Disinfection By-Products with Carcinogenicity Estimates. The DBPCAN data file, derived from data published by Woo et. al, 2002 [118] contains predicted estimates of carcinogenic potential for 209 chemicals detected in finished drinking water samples having undergone water disinfection treatment;
  - EPAFHM [77]- EPA Fathead Minnow Acute Toxicity. The EPA Fathead Minnow Acute Toxicity database was generated by the U.S. EPA Mid-Continental Ecology Division (MED) [119] for the purpose of developing an expert system to predict acute toxicity from chemical structure based on mode of action considerations. Hence, an important and unusual characteristic of this toxicity database is that the 617 tested industrial organic chemicals were expressly chosen to serve as a useful training set for development of predictive quantitative structure-activity relationships (QSARs). A second valuable aspect of this database, from a QSAR modelling perspective, is the inclusion of general mode-of-action (MOA) [120] classifications of acute toxicity response for individual chemicals derived from study results. These MOA assignments are biologically-based classifications, allowing definition of chemical similarity based upon biological activity instead of organic chemistry functional class as most commonly employed in QSAR study. MOA classifications should strengthen the scientific basis for construction of individual QSARs;
  - KIERBL [121] - EPA Estrogen Receptor Ki Binding Study (Laws et al.). This study was conducted by US EPA researchers to evaluate the validity of the rat uterine cytosolic (RUC) estrogen receptor (ER) competitive binding assay for use in the Endocrine Disruption Screening Program (EDSP) [122]. The assay measures the ability of radiolabeled 17-beta-estradiol (^3^H-E2) to bind with RUC ER in the presence of increasing concentrations of a test chemical. The data files include all published IC50 and Ki experimental results for the 50 chemicals included in Laws et al., 2006 [123], (denoted Group 1), as well as previously unpublished results for an additional 228 structurally diverse TSCA chemicals [124] for which no ER binding was observed (denoted Group 2);
  - IRISTR [125] - EPA Integrated Risk Information System (IRIS) Toxicity Review Data. IRIS is a database of human health effects that may result from exposure to various substances found in the environment. IRIS chemical files contain descriptive and quantitative information in the following categories:
    - Oral reference doses and inhalation reference concentrations (RfDs and RfCs, respectively) for chronic non-carcinogenic health effects;
    - Hazard identification, oral slope factors, and oral and inhalation unit risks for carcinogenic effects;
  - FDAMDD [126] - FDA Maximum (Recommended) Daily Dose. The US Food and Drug Administration (FDA) Center for Drug Evaluation and Research [127], Office of Pharmaceutical Science, Informatics and Computational Safety Analysis Staff's Maximum Recommended Daily Dose (FDAMDD) database contains values for over 1200 pharmaceuticals listed in Martindale [128]. Most of the maximum recommended daily dose (MRDD) values in the database were determined from pharmaceutical clinical trials that employed an oral route of exposure and daily treatments, usually for 3-12 months;
- ECETOC skin irritation [61] - 176 chemicals for which comprehensive rabbit skin irritation/corrosion data are available. The chemicals represent a range of chemical classes (acids, acrylates/methacrylates, alcohols, aldehydes, alkalis, amides, amines, brominated derivatives, chlorinated solvents, esters, ethers, fatty acids and mixtures, fragrance oils, halogenated aromatics, hydrocarbons (unsaturated), inorganics, ketones, nitriles, phenolic derivatives, S-containing compounds, soaps/surfactants, triglycerides) and different degrees of irritancy;
- Skin sensitisation (LLNA) [62] - a database that comprises local lymph node assay (LLNA) data on 211 individual chemicals, encompassing both the chemical and biologic diversity of known chemical allergens. To cover the range of relative allergenic potencies, the data set includes data on 13 extreme, 21 strong, 69 moderate, and 66 weak contact allergens, classified according to each allergen's mathematically estimated concentration of chemical required to induce a threefold stimulation index. In addition, there are also 42 chemicals that are considered to be nonsensitizers. In terms of chemical diversity, the database contains data pertaining to the chemical classes represented by aldehydes, ketones, aromatic amines, quinones, and acrylates, as well as compounds that have different reactivity mechanisms. The list of chemicals contained in the data set represents both the chemical and biologic diversity that is known to exist for chemical allergens and non-allergens;
- Bioconcentration Factor (BCF) Gold Standard Database [41] - a database holding peer reviewed high quality BCF data that is a valuable resource for development of alternative tests.

**References**

[6] **Distributed Structure-Searchable Toxicity (DSSTox) Database Network** [<http://www.epa.gov/ncct/dsstox/index.html>]

[36] **National Toxicology Program: Database Search Application** [<http://ntp-apps.niehs.nih.gov/ntp_tox/index.cfm>]

[37] **Chemical Carcinogens: Structures and Experimental Data (ISSCAN)** [<http://www.iss.it/ampp/dati/cont.php?id=233&lang=1&tipo=7>]

[38] **Ambit** [<http://ambit.sourceforge.net/>]

[41] **Search EURAS Bioconcentration Factor (BCF) Gold Standard Database** [<http://ambit.sourceforge.net/euras/>]

[59] **ECHA List of Pre-registered Substances** [<http://apps.echa.europa.eu/preregistered/pre-registered-sub.aspx>]

[60] **EC Chemical Inventories** [<http://ecb.jrc.ec.europa.eu/home.php?CONTENU=/DOCUMENTS/QSAR/INFORMATION_SOURCES/EC_CHEMICAL_INVENTORIES/>]

[61] ECETOC: **Skin irritation and corrosion Reference Chemicals data base**, *ECETOC Technical Report No. 66* 1995; European Center for Ecotoxicology and Toxicology of Chemicals, Brussels, Belgium

[62] Gerberick GF, Ryan CA, Kern PS, Schlatter H, Dearman RJ, Kimber I, Patlewicz G, Basketter DA: **Compilation of historical local lymph node assay data for the evaluation of skin sensitization alternatives**. *Dermatitis* 2005, **16**(4):157-202.

[63] **Chemical Identifier Reslover beta 3** [<http://cactus.nci.nih.gov/chemical/structure>]

[64] **ChemIDplus Advanced** [<http://chem.sis.nlm.nih.gov/chemidplus/>]

[77] **Distributed Structure-Searchable Toxicity (DSSTox) Public Database Network: EPAFHM: EPA Feathed Minnow Acute Toxicity Database** [<http://www.epa.gov/ncct/dsstox/sdf_epafhm.html>]

[79] **Distributed Structure-Searchable Toxicity (DSSTox) Public Database Network: CPDBAS: Carcinogenic Potency Database Summary Tables – All Species** [<http://www.epa.gov/NCCT/dsstox/sdf_cpdbas.html>]

[107] **MySQL** [<http://www.mysql.com/>]

[108] **CACTUS Chemical Structure Lookup Service 2008** [<http://cactus.nci.nih.gov/cgi-bin/lookup/search>]

[109] **CADD Group Cheminformatics Tools and User Services (CACTUS)** [<http://cactus.nci.nih.gov/>]

[110] **United States National Library of Medicine** [<http://www.nlm.nih.gov/>]

[111] **TOXNET – Toxicology Data Network** [<http://toxnet.nlm.nih.gov/>]

[112] **CambridgeSoft Desktop Software ChemDraw** [<http://www.cambridgesoft.com/software/ChemDraw/>]

[113] **CambridgeSoft – Life Science Enterprise Solutions** [<http://www.cambridgesoft.com/>]

[114] **European Commission Joint Research Centre: Institute for Health and Consumer Protection, Computational Toxicology Group** [<http://ecb.jrc.ec.europa.eu/qsar/about-the-group/>]

[115] **Advanced Chemistry Development ACD/Name: Generate Chemical Nomenclature from Structure** [<http://www.acdlabs.com/products/draw_nom/nom/name/>]

[116] **United States National Center for Computational Toxicology (NCCT)** [<http://www.epa.gov/ncct>]

[117] **Distributed Structure-Searchable Toxicity (DSStox) Public Database Network: DBCAN: EPA Water Disinfection By-Products with Carcinogenicity Estimates** [<http://www.epa.gov/ncct/dsstox/sdf_dbpcan.html>]

[118] Woo YT, Lai D, McLain JL, Manibusan MK, Dellarco V: **Use of Mechanism-Based Structure–Activity Relationships Analysis in Carcinogenic Potential Ranking for Drinking Water Disinfection By-Products.** *Environ Health Perspect* 2002, **110** (suppl 1):75-87. (2002).

[119] **United States Environmental Protection Agency, Mid-Continent Ecology Division** [[http://www.epa.gov/med](http://www.epa.gov/med/)]

[120] **Distributed Structure-Searchable Toxicity (DSStox) Public Database Network: Central Field Database Entry: MOA** [[http://www.epa.gov/ncct/dsstox/CentralFieldDef.html#MOA](http://www.epa.gov/ncct/dsstox/CentralFieldDef.html#MOA)]

[121] **Distributed Structure-Searchable Toxicity (DSStox) Public Database Network: KIERBL: EPA Estrogen Receptor Ki Binding Study Database** [<http://www.epa.gov/ncct/dsstox/sdf_kierbl.html>]

[122] **United States Environmental Protection Agency Endocrine Disruptor Screening Program (EDSP)** [<http://www.epa.gov/scipoly/oscpendo/>]

[123] Laws SC, Yavanhxay S, Copper RL, Eldridge JC. **Nature of the binding interaction for 50 structurally diverse chemicals with rat estrogen receptors.** *Toxicol Sci* 2006, **94**(1): 46-56.

[124] **United States Environmental Protection Agency: Summary of the Toxic Substances Control Act** [<http://www.epa.gov/lawsregs/laws/tsca.html>]

[125] **Distributed Structure-Searchable Toxicity (DSStox) Public Database Network: IRISTR: EPA Integrated Risk Information System (IRIS) Toxicity Reveiw Data** [<http://www.epa.gov/ncct/dsstox/sdf_iristr.html>]

[126] **Distributed Structure-Searchable Toxicity (DSStox) Public Database Network: FDAMDD: FDA Maximum (Recommended) Daily Dose** [<http://www.epa.gov/ncct/dsstox/sdf_fdamdd.html>]

[127] **United States Food and Drug Administration: Drugs** [<http://www.fda.gov/Drugs/default.htm>]

[128] **Wikipedia Article on “Martindale: The complete drug reference”. The Extra Pharmacopoeia (1973, 1983, and 1993) and The Physicians' Desk Reference (1995 and 1999).** [<http://en.wikipedia.org/wiki/Martindale:_The_Extra_Pharmacopoeia>]
