# Supplementary material for: Collaborative development of predictive toxicology applications
Source: J Cheminform. 2010 Aug 31;2:7. doi: 10.1186/1758-2946-2-7 (PMC2941473; doi:10.1186/1758-2946-2-7)
Supplement: Additional file 13 — Graphical Interface Description of ToxPredict Application Steps. Description of graphical user interface interactions for steps involved in execution of ToxPredict Application. [file 1758-2946-2-7-S13.DOC]

**5.13 Additional File 13: Graphical Interface Description of ToxPredict Application Steps**

*ToxPredict Step 1 - Enter/select a chemical compound*


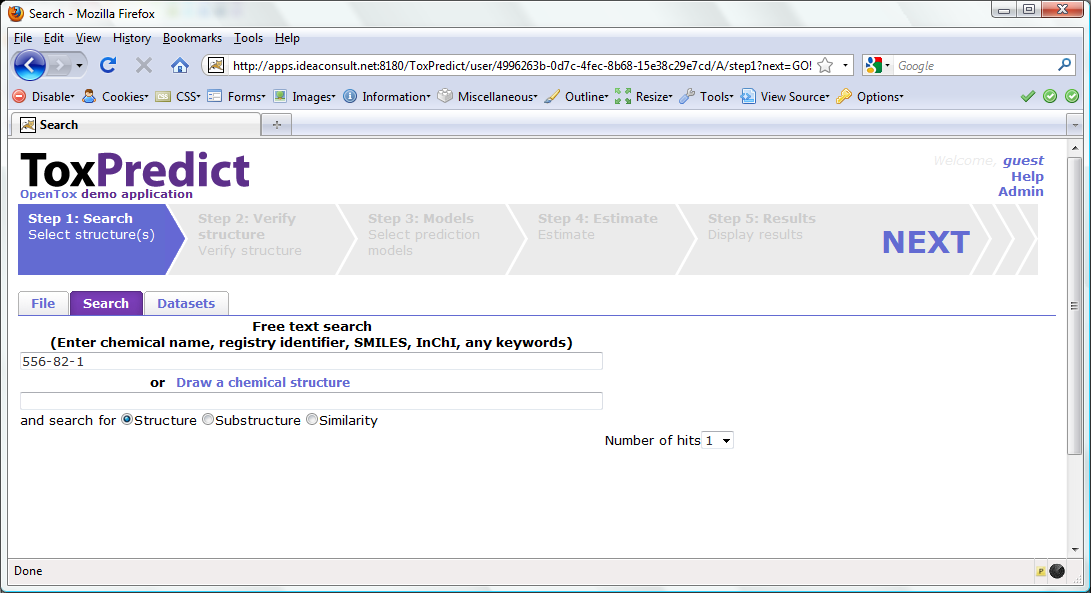


*ToxPredict Step 2 – Display selected/found structures*


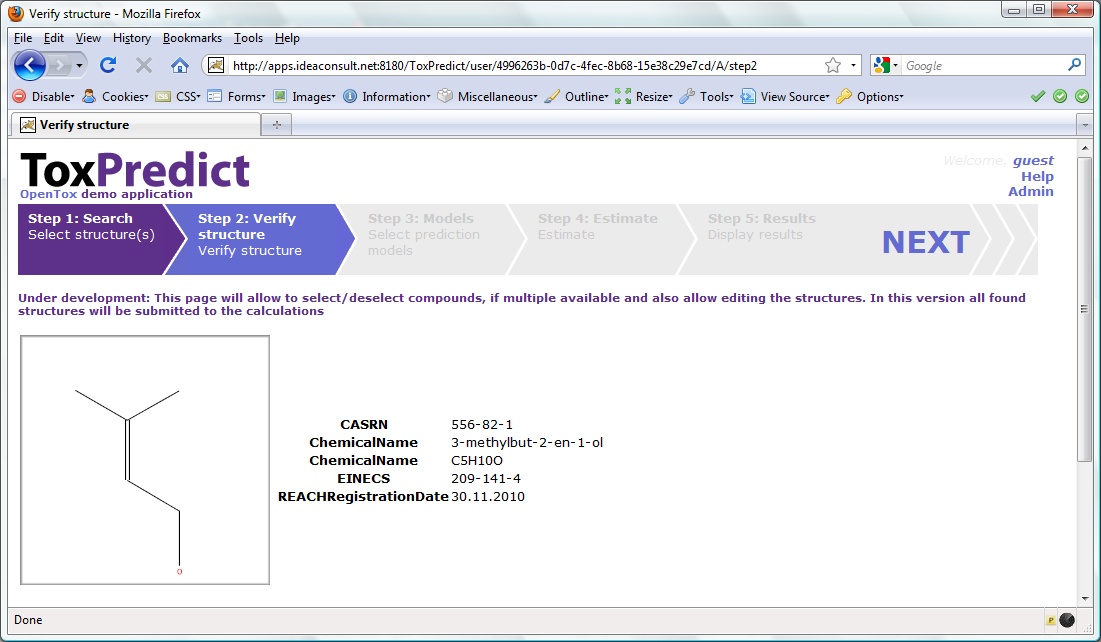


*ToxPredict Step 3 – Select models*


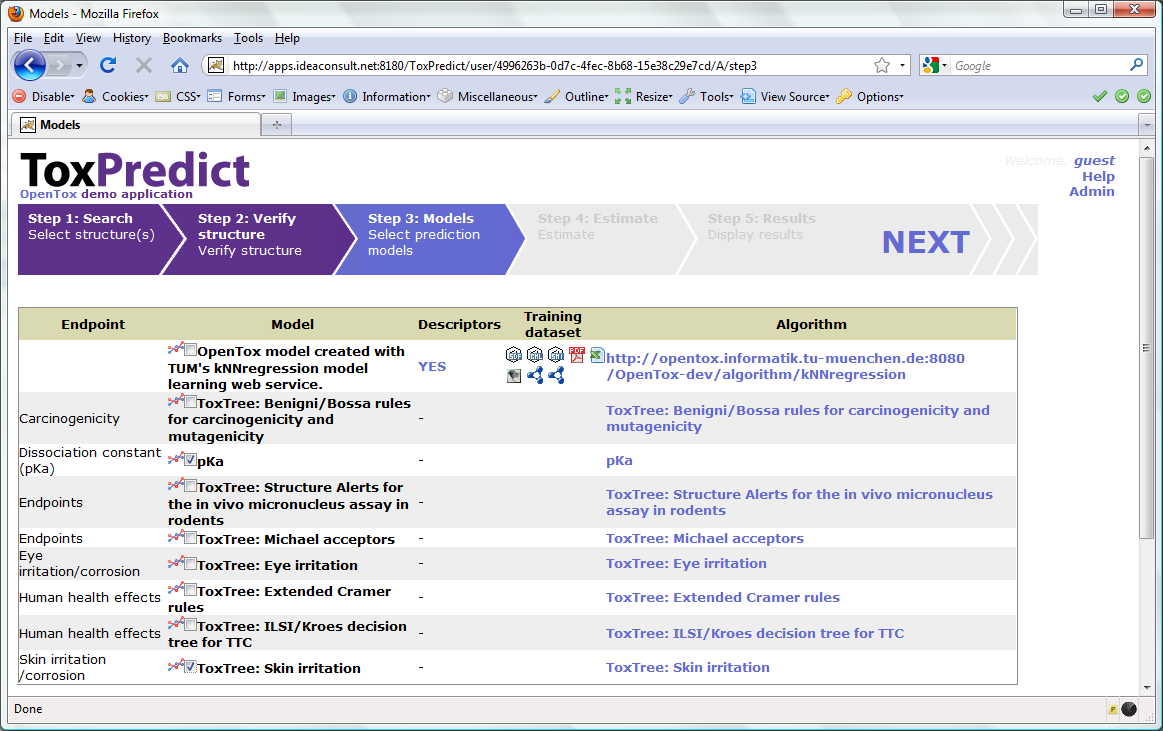


*ToxPredict Step 4 – Perform the estimation*


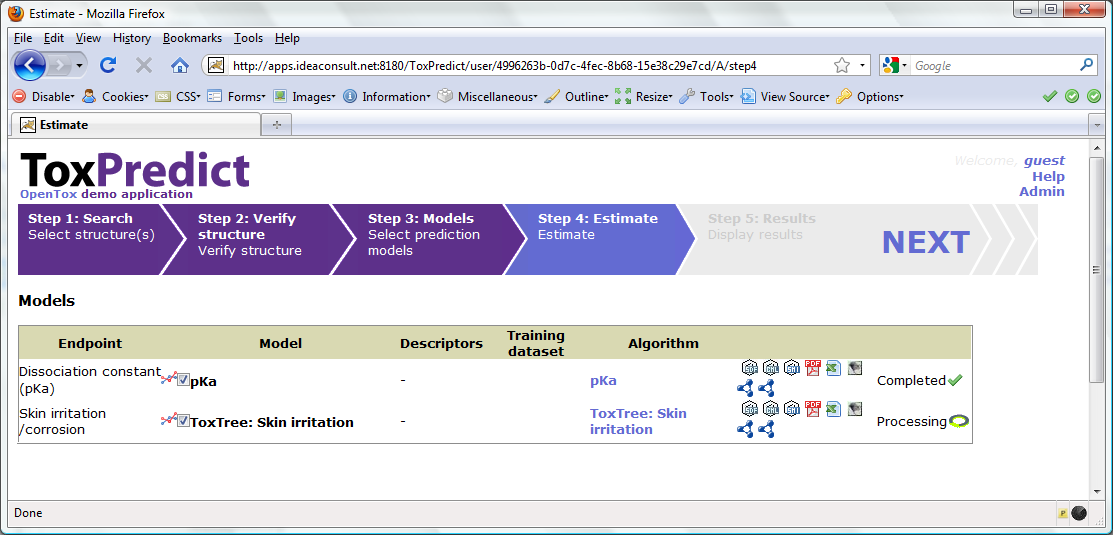


*ToxPredict Step 5 – Display the Results*

**
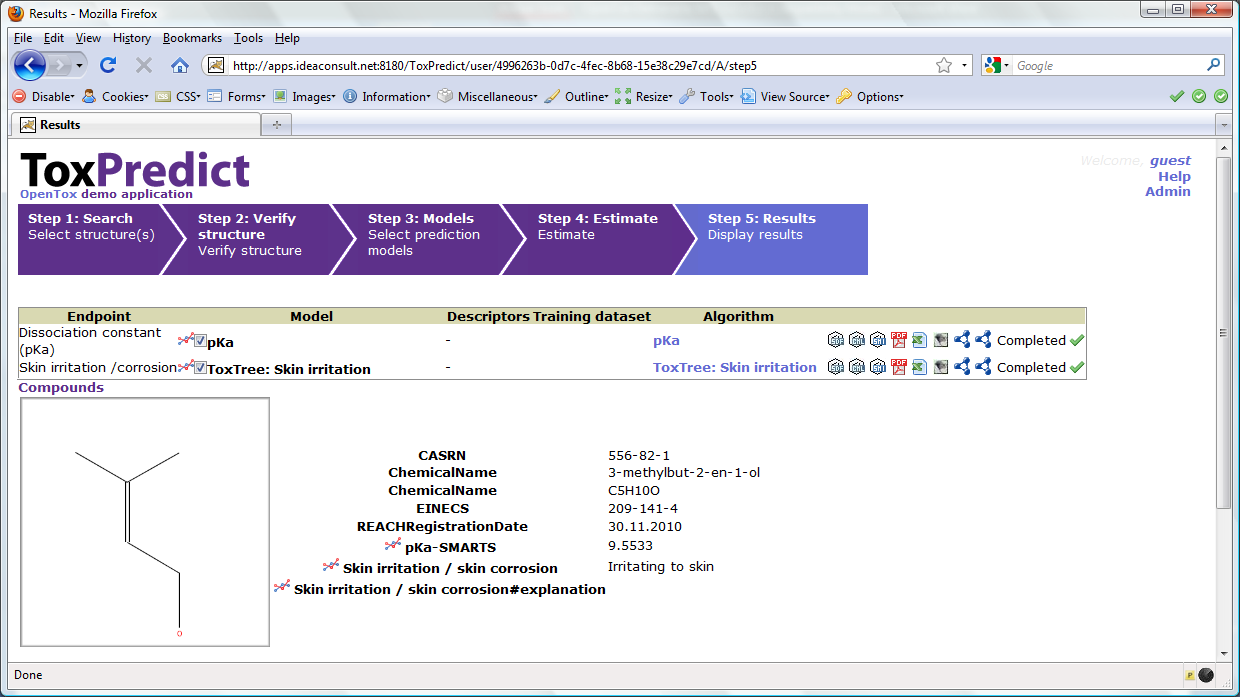
**
