# Supplementary material for: Collaborative development of predictive toxicology applications
Source: J Cheminform. 2010 Aug 31;2:7. doi: 10.1186/1758-2946-2-7 (PMC2941473; doi:10.1186/1758-2946-2-7)
Supplement: Additional file 14 — Graphical Interface Description of ToxCreate Application Steps. Description of graphical user interface interactions for steps involved in execution of ToxCreate Application. [file 1758-2946-2-7-S14.DOC]

**5.14 Additional File 14: Graphical Interface Description of ToxCreate Application Steps**

*ToxCreate Step 1 – Upload Data Set*


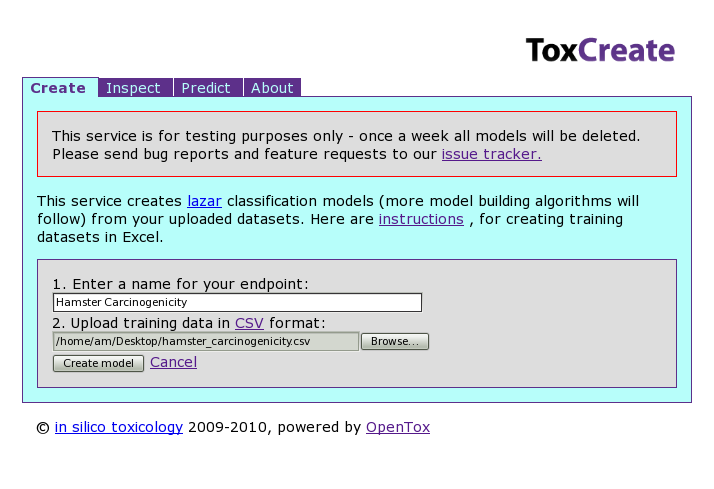


*ToxCreate Step 2 – Create and Display Model*


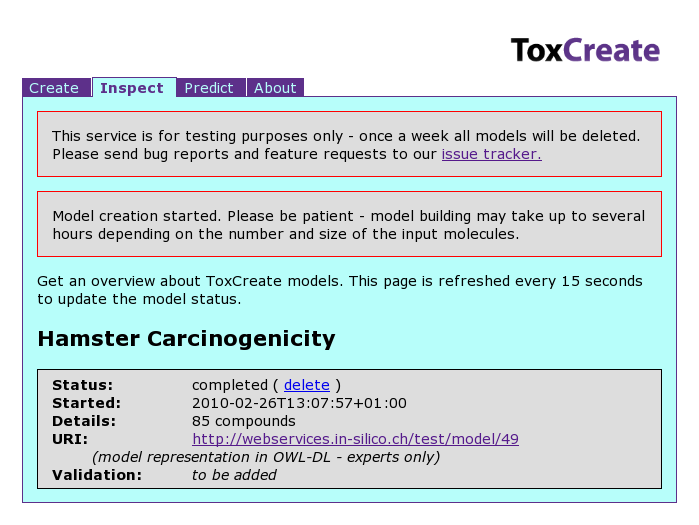


*ToxCreate Step 3 – Select and Use Model(s) for Prediction*


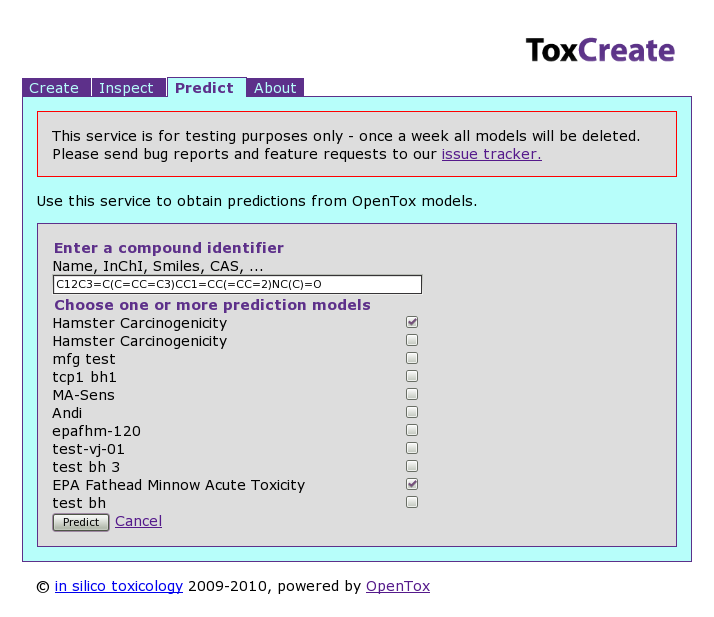


*ToxCreate Step 4 – Display Prediction* *Results*

**
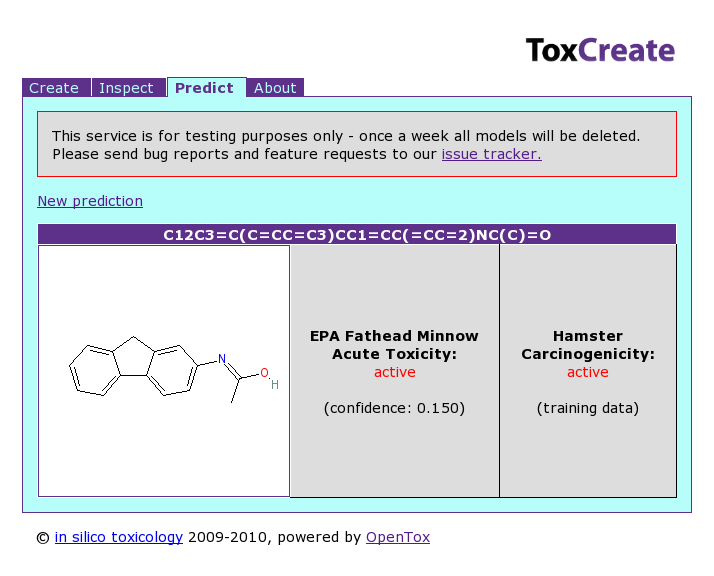
**
